# Supplementary material for: The Transcription Factor EB (TFEB) Sensitizes the Heart to Chronic Pressure Overload
Source: Int J Mol Sci. 2022 May 25;23(11):5943. doi: 10.3390/ijms23115943 (PMC9180101; doi:10.3390/ijms23115943)
Supplement: Supplementary file 1 [file ijms-23-05943-s001.zip › ijms-1729295-supplementary.pdf]

The transcription factor EB (TFEB) sensitizes the heart to chronic pressure overload

Sebastian Wundersitz[1,2], Cristina Pablo Tortola[1], Sibylle Schmidt[1], Ramon Oliveira Vidal, PhD[3,4], Melanie Kny[1], PhD, Alexander Hahn[1], Lukas Zanders[1], Hugo A. Katus[5,6], MD, Sascha Sauer[3,4], PhD, Christian Butter[7], MD, Friedrich C. Luft[1], MD, Oliver J. Müller[8,9], MD, Jens Fielitz[10,11], MD

Table S1. Morphometric analysis is indicative for a heart failure phenotype in AAV9-Tfeb but not AAV9-Luc mice following TAC.

	**AAV9-Luc**		**AAV9-Tfeb**	
	Sham	*TAC*	*Sham*	*TAC*
n	9	9	9	14
BW, g, prior to surgery	28.1 ± 0.66	27.6 ± 0.47	27.6 ± 0.43	27.8 ± 0.38
BW, g, 28 days post-surgery	27.5 ± 0.72	27.0 ± 0.50	27.3 ± 0.50	25.9 ± 0.57
HW, mg	140.7 ± 3.95	194.0 ± 10.05***	148.0 ± 4.90	239.61 ± 8.12*** #
Lung weight, mg	142.7 ± 3.59	169.0 ± 12.60	146.1 ± 3.46	316.1 ± 30.34** #
Liver weight, mg	1265.4 ± 41.15	1301.8 ± 65.72	1280.3 ± 42.59	1053.0 ± 51.29** #
TL, mm	17.02 ± 0.09	16.98 ± 0.06	17.00 ± 0.07	16.95 ± 0.05
HW/TL, mg/mm	8.26 ± 0.20	11.41 ± 0.57***	8.71 ± 0.30	14.13 ± 0.47*** #

Data are shown as mean ± SEM. HW indicates heart weight; BW, body weight; TL, tibia length. *p<0.05, **p<0.01, ***p<0.001 compared to corresponding sham mice. †p<0.05 AAV9-Luc sham compared AAV9-Tfeb sham mice. #p<0.05 AAV9-Luc TAC compared to AAV9-Tfeb TAC mice.

Table S2. Echocardiographic analysis shows that cardiomyocyte-specific TFEB overexpression leads to heart failure in response to pressure-overload.

	**AAV9-Luc**		**AAV9-Tfeb**	
	Sham	*TAC*	*Sham*	*TAC*
n	9	9	9	14
HR, bpm	496.2 ± 14.5	503.9 ± 19.99	462.3 ± 11.8	521.5 ± 10.69**
Morphology				
LVEDD, mm	3.83 ± 0.09	4.08 ± 0.22	4.12 ± 0.09†	5.16 ± 0.19$^{***, \#\#\#}$
LVESD, mm	2.54 ± 0.11	3.20 ± 0.21*	2.94 ± 0.14†	4.62 ± 0.25$^{***, \#\#\#}$
IVSthd, mm	0.77 ± 0.02	1.02 ± 0.03***	0.73 ± 0.02	0.89 ± 0.03$^{**, \#}$
IVSths, mm	1.16 ± 0.02	1.32 ± 0.05*	1.03 ± 0.05†	1.09 ± 0.04$^{\#\#}$
PWthd, mm	0.77 ± 0.02	1.01 ± 0.03***	0.74 ± 0.03	0.92 ± 0.04**
PWths, mm	1.15 ± 0.03	1.36 ± 0.07*	1.07 ± 0.06	1.10 ± 0.04$^{\#\#}$
Systolic function				
FS, %	33.84 ± 1.50	21.68 ± 2.46**	29.02 ± 2.51	10.17 ± 1.36$^{***, \#\#\#}$
LVEF, %	64.6 ± 1.93	52.3 ± 5.54	56.34 ± 2.55†	22.41 ± 2.92$^{***, \#\#\#}$
SV, µl	27.3 ± 1.49	27.6 ± 2.23	26.5 ± 1.13	19.4 ± 1.62$^{***, \#\#\#}$
Diastolic function				
Mitral E/A ratio	1.32 ± 0.162	1.57 ± 0.1*	1.57 ± 0.38	2.12 ± 0.36$^{*, \#}$
Mitral E velocity, mm/s	649.7 ± 52.9	770.1 ± 196.2	676.7 ± 114.1	600.7 ± 194.8
Mitral A velocity, mm/s	500.8 ± 72.3	487.9 ± 109.5	461.4 ± 152.9	283.8 ± 115.4$^{\#}$
E/E`	27.3 ± 4.7	37.1 ± 10.5	28.8 ± 4.3	38.7 ± 6.3
IVRT, ms	16.7 ± 3.2	15.6 ± 3.9	18.5 ± 2.2	17.8 ± 3.5
IVCT, ms	19.8 ± 4.2	23.7 ± 8.7	20.6 ± 6.4	23.8 ± 9.5
ET, ms	38.8 ± 6.3	38.4 ± 9.0	36.8 ± 5.0	38.9 ± 8.9

ET indicates ejection time; HR, heart rate; IVCT, isovolumic contraction time; IVRT, isovolumic relaxation time; IVSths, interventricular septal wall thickness at systole; IVSthd, interventricular septal wall thickness at diastole; LVEDD, left ventricular enddiastolic diameter; LVESD, left ventricular endsystolic diameter; PWths, posterior wall thickness in systole; PWthd, posterior wall thickness in diastole; SV, left ventricular stroke volume; FS, left ventricular fractional shortening, LVEF, left ventricular ejection fraction. $^{*}p<0.05$, $^{**}p<0.01$, $^{***}p<0.001$ compared to corresponding sham operated mice; †p<0.05 AAV9-Luc sham compared to AAV9-Tfeb sham mice. Data are expressed as mean ± SEM. $^{\#}p<0.05$, $^{\#\#}p<0.01$, $^{\#\#\#}p<0.001$ AAV9-Luc TAC compared to AAV9-Tfeb TAC mice.

Table S3. Sequencing results for each experimental group.

Name	Initial Reads	Uniquely Mapped Reads	Uniquely Mapped Reads (%)
**AAV9-Luc Sham1**	33.10 M	25.58 M	77.26
**AAV9-Luc Sham2**	26.45 M	20.35 M	76.95
**AAV9-Luc Sham3**	29.77 M	23.29 M	78.22
**AAV9-Tfeb Sham1**	26.43 M	20.56 M	77.80
**AAV9-Tfeb Sham2**	27.96 M	21.81 M	78.00
**AAV9-Tfeb Sham3**	31.03 M	23.86 M	76.90
**AAV9-Luc TAC1**	28.05 M	22.92 M	81.72
**AAV9-Luc TAC2**	29.38 M	23.33 M	79.43
**AAV9-Luc TAC3**	31.84 M	24.69 M	77.54
**AAV9-Luc TAC4**	34.98 M	28.48 M	81.44
**AAV9-Tfeb TAC1**	46.88 M	38.12 M	81.32
**AAV9-Tfeb TAC2**	32.51 M	25.75 M	79.21
**AAV9-Tfeb TAC3**	35.31 M	27.99 M	79.26

Overview of total reads and uniquely mapped reads generated by RNA-Sequencing for each animal included. M indicates 1 Million reads.

Table S4. RNA sequencing results

Please refer to .xls Table.

Table S5. KEGG pathway analysis of all DEG in hearts of AAV9-Luc TAC but not AAV9-Tfeb TAC treated mice

Term	Count	%	PValue	Genes	List Total	Pop Hits	Pop Total	Fold Enrichment	Bonferroni	Benjamini	FDR
mmu05417:Lipid and atherosclerosis	20	3,571	3,6E-05	CCL12, CD40, HSPA5, APOA1, POU2F2, SOD2, PYCARD, NRAS, AKT2, TRP53, IL1B, CASP1, CCL2, NLRP3, CD36, TLR6, PLCB1, CALM1, LDLR, MYD88	276	216	8941	3,000	0,010	0,010	0,010
mmu04621:NOD-like receptor signaling pathway	18	3,214	2,7E-04	GSDMD, NLRP1B, CCL12, NLRP1A, CARD9, TNFAIP3, OAS1A, OAS1G, PYCARD, AIM2, NAIP6, IL1B, CASP1, CCL2, NLRP3, PLCB1, MYD88, BIRC3	276	211	8941	2,764	0,075	0,033	0,030
mmu04064:NF-kappa B signaling pathway	12	2,143	3,7E-04	CD40, PRKCB, IL1B, LTA, TNFAIP3, TNFRSF11A, BCL2A1D, PTGS2, BCL2A1B, CARD11, MYD88, BIRC3	276	105	8941	3,702	0,102	0,033	0,030
mmu04110:Cell cycle	13	2,321	4,5E-04	BUB1B, TTK, CDC25C, CCNA2, CDC20, WEE1, CCNB1, CCNE1, TRP53, E2F1, MCM3, E2F2, BUB1	276	125	8941	3,369	0,124	0,033	0,030
mmu04625:C-type lectin receptor signaling pathway	12	2,143	6,4E-04	PYCARD, CLEC4B1, NRAS, PAK1, CCL22, IL1B, AKT2, CARD9, CASP1, NLRP3, CALM1, PTGS2	276	112	8941	3,471	0,171	0,038	0,034
mmu04060:Cytokine-cytokine receptor interaction	20	3,571	1,6E-03	TNFSF18, CCL24, CCL12, CD40, CCL22, MPL, OSM, LIF, INHBA, TNFRSF11A, BMP6, IL27RA, IL1RL2, IL1B, LTA, CCL2, CD27, CCR7, TNFSF8, IL7R	276	292	8941	2,219	0,385	0,081	0,074
mmu05132:Salmonella infection	18	3,214	2,0E-03	GSDMD, MLKL, RHOG, RHOH, DYNLL1, PYCARD, TUBB6, PAK1, TUBB5, NAIP6, AKT2, IL1B, CASP1, NLRP3, TLR6, MYD88, S100A10, BIRC3	276	253	8941	2,305	0,454	0,086	0,079
mmu05200:Pathways in cancer	30	5,357	2,5E-03	GSTP2, EPAS1, FGF1, PTGS2, EGFR, RASGRP4, DLL4, NRAS, GNGT2, AKT2, CTNNA1, STAT4, E2F1, E2F2, PMAIP1, LAMB2, PRKCB, WNT9B, CCNA2, RAD51, GSTA3, CCNE1, TRP53, GSTA1, CKS2, BIRC5, IL7R, CALM1, PLCB1, BIRC3	276	543	8941	1,790	0,517	0,089	0,082
mmu01524:Platinum drug resistance	9	1,607	3,1E-03	TOP2A, GSTP2, GSTA3, AKT2, TRP53, GSTA1, PMAIP1, BIRC5, BIRC3	276	80	8941	3,644	0,598	0,089	0,082
mmu04210:Apoptosis	12	2,143	3,1E-03	PARP3, NRAS, AKT2, TRP53, PRF1, PMAIP1, BIRC5, CTSW, CTSF, BCL2A1D, BCL2A1B, BIRC3	276	136	8941	2,858	0,599	0,089	0,082
mmu04662:B cell receptor signaling pathway	9	1,607	3,3E-03	NRAS, IFITM1, PIRA2, PRKCB, AKT2, PIK3AP1, CARD11, CD22, LILRA5	276	81	8941	3,599	0,626	0,089	0,082
mmu00270:Cysteine and methionine metabolism	7	1,250	5,5E-03	LDHB, MDH1, GOT1, PSAT1, KYAT3, MAT2B, BCAT2	276	53	8941	4,279	0,802	0,134	0,123
mmu05214:Glioma	8	1,429	7,5E-03	NRAS, PRKCB, AKT2, TRP53, E2F1, E2F2, CALM1, EGFR	276	74	8941	3,502	0,891	0,146	0,133
mmu05222:Small cell lung cancer	9	1,607	7,7E-03	LAMB2, CCNE1, AKT2, TRP53, E2F1, CKS2, E2F2, PTGS2, BIRC3	276	93	8941	3,135	0,898	0,146	0,133
mmu05144:Malaria	7	1,250	7,8E-03	CD40, CCL12, IL1B, CCL2, ACKR1, CD36, MYD88	276	57	8941	3,978	0,901	0,146	0,133
mmu04668:TNF signaling pathway	10	1,786	7,9E-03	CCL12, MLKL, IL1B, AKT2, LTA, LIF, CCL2, TNFAIP3, PTGS2, BIRC3	276	113	8941	2,867	0,904	0,146	0,133
mmu00620:Pyruvate metabolism	6	1,071	1,1E-02	LDHB, ADH1, MDH1, ME3, ACSS1, ACACB	276	44	8941	4,417	0,960	0,188	0,171
mmu04936:Alcoholic liver disease	11	1,964	1,2E-02	ADH1, ACADVL, IL1B, AKT2, C5AR1, PRKAG1, MLYCD, LPIN1, ACACB, MYD88, C2	276	141	8941	2,527	0,968	0,189	0,173
mmu04710:Circadian rhythm	5	0,893	1,3E-02	PER2, PER1, CRY2, BHLHE40, PRKAG1	276	30	8941	5,399	0,978	0,198	0,181
mmu00640:Propanoate metabolism	5	0,893	1,4E-02	BCKDHA, LDHB, MLYCD, ACSS1, ACACB	276	31	8941	5,225	0,986	0,211	0,193
mmu05164:Influenza A	12	2,143	1,8E-02	PYCARD, H2-DMB2, CCL12, PRKCB, IL1B, AKT2, CASP1, CCL2, OAS1A, NLRP3, MYD88, OAS1G	276	173	8941	2,247	0,995	0,249	0,227
mmu05208:Chemical carcinogenesis - reactive oxygen species	14	2,500	1,9E-02	EPHX2, ATP5A1, SDHC, COX6C, SOD2, COX7C, EGFR, NRAS, GSTA3, AKT2, GSTA1, NDUFAB1, NDUFS2, NDUFV1	276	222	8941	2,043	0,997	0,255	0,233
mmu05145:Toxoplasmosis	9	1,607	2,0E-02	H2-DMB2, CD40, SOCS1, LAMB2, AKT2, TLR11, LDLR, MYD88, BIRC3	276	110	8941	2,650	0,997	0,255	0,233
mmu00982:Drug metabolism - cytochrome P450	7	1,250	2,2E-02	ADH1, HPGDS, GSTP2, GSTA3, GSTA1, FMO1, FMO2	276	71	8941	3,194	0,998	0,261	0,238
mmu05218:Melanoma	7	1,250	2,3E-02	NRAS, AKT2, TRP53, E2F1, E2F2, FGF1, EGFR	276	72	8941	3,150	0,999	0,261	0,238
mmu05223:Non-small cell lung cancer	7	1,250	2,3E-02	NRAS, PRKCB, AKT2, TRP53, E2F1, E2F2, EGFR	276	72	8941	3,150	0,999	0,261	0,238
mmu05169:Epstein-Barr virus infection	14	2,500	2,6E-02	CD40, H2-M3, TNFAIP3, OAS1A, OAS1G, CCNA2, H2-DMB2, CCNE1, AKT2, TRP53, E2F1, E2F2, CD247, MYD88	276	231	8941	1,963	1,000	0,280	0,255
mmu05163:Human cytomegalovirus infection	15	2,679	2,7E-02	CCL12, PRKCB, H2-M3, PTGS2, EGFR, NRAS, GNGT2, AKT2, TRP53, IL1B, E2F1, CCL2, E2F2, PLCB1, CALM1	276	256	8941	1,898	1,000	0,280	0,255
mmu04964:Proximal tubule bicarbonate reclamation	4	0,714	2,9E-02	MDH1, ATP1A3, ATP1B2, CAR4	276	22	8941	5,890	1,000	0,283	0,258
mmu05161:Hepatitis B	11	1,964	2,9E-02	CCNA2, NRAS, CCNE1, PRKCB, AKT2, TRP53, STAT4, E2F1, E2F2, BIRC5, MYD88	276	163	8941	2,186	1,000	0,283	0,258
mmu05133:Pertussis	7	1,250	3,1E-02	PYCARD, IL1B, CASP1, NLRP3, CALM1, MYD88, C2	276	77	8941	2,945	1,000	0,285	0,260
mmu05143:African trypanosomiasis	5	0,893	3,1E-02	PRKCB, IL1B, APOA1, PLCB1, MYD88	276	39	8941	4,153	1,000	0,285	0,260
mmu05215:Prostate cancer	8	1,429	3,3E-02	NRAS, GSTP2, CCNE1, AKT2, TRP53, E2F1, E2F2, EGFR	276	99	8941	2,618	1,000	0,287	0,262
mmu05012:Parkinson disease	15	2,679	3,3E-02	UBA7, HSPA5, ATP5A1, SDHC, UBE2L6, COX6C, COX7C, TUBB6, TUBB5, TRP53, NDUFAB1, NDUFS2, CALM1, NDUFV1, SLC18A2	276	264	8941	1,841	1,000	0,287	0,262
mmu05170:Human immunodeficiency virus 1 infection	14	2,500	3,4E-02	TRIM30D, PRKCB, H2-M3, CDC25C, BST2, NRAS, PAK1, WEE1, CCNB1, GNGT2, AKT2, CD247, CALM1, MYD88	276	240	8941	1,890	1,000	0,287	0,262
mmu05219:Bladder cancer	5	0,893	3,6E-02	NRAS, TRP53, E2F1, E2F2, EGFR	276	41	8941	3,951	1,000	0,298	0,272
mmu05142:Chagas disease	8	1,429	3,9E-02	CCL12, IL1B, AKT2, CCL2, CD247, TLR6, PLCB1, MYD88	276	103	8941	2,516	1,000	0,312	0,285
mmu05226:Gastric cancer	10	1,786	4,2E-02	NRAS, CCNE1, AKT2, TRP53, CTNNA1, E2F1, WNT9B, E2F2, FGF1, EGFR	276	150	8941	2,160	1,000	0,319	0,291
mmu05225:Hepatocellular carcinoma	11	1,964	4,2E-02	NRAS, GSTP2, GSTA3, PRKCB, AKT2, TRP53, GSTA1, E2F1, WNT9B, E2F2, EGFR	276	174	8941	2,048	1,000	0,319	0,291
mmu05206:MicroRNAs in cancer	16	2,857	4,7E-02	HDAC5, PRKCB, CDCA5, KIF23, CDC25C, PTGS2, EGFR, NRAS, MARCKS, SOCS1, CCNE1, TRP53, STMN1, E2F1, TIMP3, E2F2	276	303	8941	1,711	1,000	0,342	0,312
mmu04970:Salivary secretion	7	1,250	4,9E-02	PRKCB, LYZ1, ATP1A3, ATP1B2, CALM1, PLCB1, PRKG1	276	86	8941	2,637	1,000	0,342	0,312
mmu04540:Gap junction	7	1,250	4,9E-02	NRAS, TUBB6, TUBB5, PRKCB, PLCB1, EGFR, PRKG1	276	86	8941	2,637	1,000	0,342	0,312

Table S6. KEGG pathway analysis of all DEG in hearts of AAV9-Luc and AAV9-Tfeb TAC treated mice

Term	Count	%	PValue	Genes	List Tota	Pop Hits	Pop Tota	Fold Enrichmen	Bonferror	Benjamir	FDR
mmu04512:ECM-receptor interaction	26	1,548	6,2E-08	ITGB5, SDC4, TNC, LAMC2, HMMR, THBS2, THBS4, THBS3, SPP1, TNR, FN1, GP6, COL1A1, COL1A2, COL4A2, COL4A1, COL6A2, COL6A1, COL4A6, SDC1, COL6A6, ITGA5, COL9A2, FREM1, CD44, ITGA9	778	88	8941	3,395	2,0E-05	2,0E-05	1,7E-05
mmu04974:Protein digestion and absorption	27	1,607	1,2E-06	COL15A1, COL16A1, COL14A1, ELN, COL11A2, COL12A1, PRCP, SLC3A2, SLC38A2, SLC36A2, MME, SLC16A10, COL1A1, COL3A1, COL1A2, COL4A2, COL5A1, COL4A1, COL6A2, COL5A3, COL5A2, COL6A1, COL4A6, COL8A2, COL8A1, COL6A6, COL9A2	778	108	8941	2,873	3,9E-04	1,3E-04	1,1E-04
mmu04510:Focal adhesion	40	2,381	1,2E-06	SHC4, ITGB5, TNC, LAMC2, THBS2, THBS4, ACTB, MYLK3, THBS3, MYLK, ACTG1, MYLK4, RAP1B, CDC42, PDGFC, SPP1, TNR, FLNC, VASP, CAV3, ACTN1, FN1, IGF1, ACTN4, COL1A1, DIAPH1, COL1A2, COL4A2, COL4A1, COL6A2, COL6A1, COL4A6, ZYX, BCL2, COL6A6, ITGA5, COL9A2, TLN1, MET, ITGA9	778	201	8941	2,287	4,0E-04	1,3E-04	1,1E-04
mmu05205:Proteoglycans in cancer	40	2,381	2,1E-06	CD63, CDKN1A, WNT2B, ITGB5, SDC4, CAMK2A, HIF1A, ACTB, ACTG1, CDC42, RRAS, MYC, PLCG2, FLNC, FZD1, TGFB2, TGFB1, CAV3, LUM, PTCH1, MMP2, STAT3, IGF2, PLAUR, GAB1, FN1, MSN, RRAS2, IGF1, DCN, COL1A1, COL1A2, CTTN, SDC1, HCLS1, ITGA5, TLR4, MET, CD44, HBEGF	778	205	8941	2,242	6,6E-04	1,7E-04	1,4E-04
mmu00280:Valine, leucine and isoleucine degradation	18	1,071	3,9E-06	MCCC2, ACAA2, MCCC1, BCKDHB, ACADSB, AACS, HADHB, HADHA, ALDH6A1, PCCA, ALDH2, IVD, AOX3, DBT, AOX1, ACADM, HADH, ALDH9A1	778	57	8941	3,629	1,2E-03	2,5E-04	2,1E-04
mmu04010:MAPK signaling pathway	49	2,917	1,3E-05	FLT3, HSPB1, FGF6, RPS6KA5, MYC, RPS6KA1, MAP3K5, DUSP5, MAP4K2, DUSP2, PLA2G4B, PLA2G4A, CACNA2D2, RRAS2, FOS, TGFBR1, TNFRSF1A, EREG, TGFBR2, DUSP7, CACNB1, CACNB2, RAPGEF2, MET, ATF4, MAX, RASGRP2, CACNA1H, RELB, RAP1B, CDC42, MAPK7, RRAS, NTF3, PDGFC, CD14, FLNC, TGFB2, TGFB1, ANGPT1, TGFB3, IGF2, IGF1, GNG12, FGF16, PTPN7, MAP3K13, MAP3K14, FGF10	778	294	8941	1,915	4,0E-03	5,9E-04	4,9E-04
mmu05414:Dilated cardiomyopathy	23	1,369	1,3E-05	TGFB2, TGFB1, ITGB5, TGFB3, TPM3, ATP2A2, CACNA2D2, ADRB1, IGF1, ADCY7, ACTB, ADCY6, ACTG1, CACNB1, CACNB2, DES, PLN, LMNA, TNNI3, ITGA5, MYH6, ITGA9, MYH7	778	94	8941	2,812	4,1E-03	5,9E-04	4,9E-04
mmu04933:AGE-RAGE signaling pathway in diabetic complications	23	1,369	4,2E-05	TGFB2, TGFB1, TGFB3, MMP2, PRKCD, SERPINE1, STAT3, FN1, F3, TGFBR1, TGFBR2, ICAM1, COL1A1, CDC42, DIAPH1, IL6, COL3A1, COL1A2, COL4A2, COL4A1, COL4A6, BCL2, PLCG2	778	101	8941	2,617	1,4E-02	1,7E-03	1,4E-03
mmu04810:Regulation of actin cytoskeleton	38	2,262	6,5E-05	CHRM2, ITGB5, ARPC1B, LPAR1, IQGAP3, ACTB, MYLK3, MYLK, ACTG1, MYLK4, GNA13, CDC42, FGF6, RRAS, CFL1, PDGFC, MYH11, ACTR3, ACTN1, F2R, FN1, MSN, RRAS2, ARPC4, ACTN4, ARPC5, BAIAP2, GNG12, ENAH, FGF16, DIAPH1, DIAPH3, ARPC2, ARPC3, ITGA5, PFN1, FGF10, ITGA9	778	220	8941	1,985	2,1E-02	2,1E-03	1,7E-03
mmu05100:Bacterial invasion of epithelial cells	19	1,131	6,6E-05	ACTR3, SHC4, CAV3, ARPC1B, GAB1, FN1, ARPC4, ARPC5, ACTB, DNM1, ACTG1, CDC42, ARPC2, CTTN, ARPC3, ELMO1, HCLS1, ITGA5, MET	778	76	8941	2,873	2,1E-02	2,1E-03	1,7E-03
mmu04151:PI3K-Akt signaling pathway	54	3,214	7,8E-05	CHRM2, CDKN1A, ITGB5, FLT3, TNC, LAMC2, GHR, FGF6, CREB3L1, MYC, TNR, F2R, OSMR, YWHAZ, EREG, RBL2, CREB3, COL4A2, COL4A1, COL6A2, COL6A1, IL3RA, COL4A6, COL6A6, ITGA5, TLR4, MET, ATF4, ITGA9, CREB5, LPAR1, IL4RA, FOXO3, THBS2, THBS4, THBS3, RXRA, NTF3, PDGFC, SPP1, EIF4EBP1, ANGPT1, IGF2, FN1, IGF1, GNG12, COL1A1, FGF16, IL6, COL1A2, PPP2R2C, BCL2, COL9A2, FGF10	778	359	8941	1,729	2,5E-02	2,1E-03	1,7E-03
mmu05410:Hypertrophic cardiomyopathy	21	1,250	8,2E-05	TGFB2, TGFB1, ACE, ITGB5, TGFB3, TPM3, ATP2A2, CACNA2D2, IGF1, ACTB, ACTG1, CACNB1, CACNB2, IL6, DES, LMNA, TNNI3, ITGA5, MYH6, ITGA9, MYH7	778	91	8941	2,652	2,6E-02	2,1E-03	1,7E-03
mmu04145:Phagosome	33	1,964	8,5E-05	COLEC12, RAB5C, TFRC, ITGB5, NCF4, H2-K1, THBS2, CORO1A, THBS4, ACTB, THBS3, ACTG1, MRC2, TUBA1C, TUBA1B, TUBA1A, TUBB3, H2-DMB1, ATP6V1H, ATP6V0A4, CD14, CYBA, FCGR1, DYNC1LI1, TUBB2B, TUBB2A, FCGR4, CANX, ATP6V1B2, ITGA5, RAB7B, TLR4, H2-AB1	778	182	8941	2,084	2,7E-02	2,1E-03	1,7E-03
mmu04666:Fc gamma R-mediated phagocytosis	21	1,250	9,6E-05	ACTR3, VASP, MARCKSL1, MYO10, PLA2G4B, SPHK1, ARPC1B, PRKCD, PLA2G4A, ARPC4, GAB2, ARPC5, FCGR1, CDC42, ARPC2, FCGR4, ARPC3, CFL1, PLCG2, PLPP2, ARF6	778	92	8941	2,623	3,0E-02	2,2E-03	1,8E-03
mmu05132:Salmonella infection	41	2,440	1,4E-04	RAB5C, ARPC1B, ACTB, ACTG1, CYTH3, CDC42, FYCO1, TUBA1C, TUBA1B, TUBA1A, CYTH4, RRAS, TUBB3, KIF5C, MYC, KIF5B, CASP4, BAK1, CD14, FLNC, ACTR3, ANXA2, AHNAK2, ARPC4, FOS, ARPC5, TNFRSF1A, DYNC1LI1, IL6, TUBB2B, TUBB2A, ARPC2, ARPC3, RHOJ, BCL2, ELMO1, PFN1, RAB7B, TLR4, ARL8A, ARF6	778	253	8941	1,862	4,3E-02	2,9E-03	2,4E-03
mmu04611:Platelet activation	25	1,488	1,4E-04	RASGRP2, ADCY7, ACTB, MYLK3, ADCY6, MYLK, ACTG1, GNAI2, MYLK4, GNA13, RAP1B, P2RY1, PLCG2, VASP, FCER1G, PLA2G4B, F2R, PLA2G4A, GP6, COL1A1, APBB1IP, COL3A1, COL1A2, P2RX1, TLN1	778	124	8941	2,317	4,5E-02	2,9E-03	2,4E-03
mmu05200:Pathways in cancer	73	4,345	1,5E-04	CDKN1A, WNT2B, FLT3, SLC2A1, LAMC2, BBC3, FGF6, RPS6KA5, MYC, RASSF5, IL13RA1, IL15RA, IL15, TPM3, IFNGR1, MMP2, F2R, MITF, FOS, TGFBR1, TGFBR2, RUNX1, CSF2RB2, MSH2, COL4A2, TRAF3, COL4A1, IL3RA, COL4A6, RARB, MET, PTGER4, CEBPA, MAX, CAMK2A, LPAR1, IL4RA, GSTT2, GSTT1, CSF2RB, RASGRP2, HIF1A, CSF2RA, ADCY7, ADCY6, GNAI2, GNA13, CDC42, RXRA, PLCG2, E2F3, BAK1, BID, RUNX1T1, EGLN1, FZD1, TGFB2, GSTM1, TGFB1, TGFB3, PTCH1, ZBTB16, STAT3, IGF2, FN1, IGF1, GNG12, FGF16, IL6, BCL2, CALM2, GSTM7, FGF10	778	543	8941	1,545	4,8E-02	2,9E-03	2,4E-03
mmu04066:HIF-1 signaling pathway	23	1,369	2,8E-04	EGLN1, CDKN1A, ANGPT1, TFRC, IFNGR1, CAMK2A, SERPINE1, STAT3, SLC2A1, IGF1, ENO1, HIF1A, IL6, NPPA, BCL2, EIF4EBP1, PLCG2, TIMP1, LTBR, ALDOB, TLR4, PFKM, PFKP	778	114	8941	2,319	8,5E-02	4,7E-03	3,9E-03
mmu04921:Oxytocin signaling pathway	28	1,667	2,8E-04	CDKN1A, CAMK2A, ADCY7, ACTB, MYLK3, ADCY6, MYLK, ACTG1, GNAI2, MYLK4, MAPK7, NPPA, KCNJ2, KCNJ3, KCNJ5, KCNJ6, KCNJ12, PLA2G4B, KCNJ14, PLA2G4A, CACNA2D2, FOS, CACNB1, GNAO1, RCAN1, CACNB2, MYL6, CALM2	778	153	8941	2,103	8,6E-02	4,7E-03	3,9E-03
mmu04060:Cytokine-cytokine receptor interaction	44	2,619	3,9E-04	CX3CR1, CNTFR, CTF1, IL4RA, IL20RB, TNFRSF11B, CSF2RB, CXCR6, CSF2RA, CX3CL1, TNFSF13B, CXCL16, GHR, CCL8, IL21R, RELT, IL13RA1, GDF10, IL15RA, TGFB2, XCR1, TGFB1, TNFRSF12A, GDF15, IL15, IFNGR1, TGFB3, IL1R2, TNFRSF19, INHBB, LIFR, GDF6, OSMR, TNFRSF1B, TGFBR1, IL17RA, TNFRSF1A, TGFBR2, CSF2RB2, IL6, IL3RA, ACKR4, LTBR, CRLF2	778	292	8941	1,732	1,2E-01	6,2E-03	5,2E-03
mmu05146:Amoebiasis	21	1,250	7,9E-04	TGFB2, TGFB1, RAB5C, TGFB3, ACTN1, IL1R2, FN1, HSPB1, LAMC2, ACTN4, COL1A1, IL6, COL3A1, COL1A2, COL4A2, SERPINB6B, COL4A1, COL4A6, CD14, RAB7B, TLR4	778	107	8941	2,255	2,3E-01	1,2E-02	1,0E-02
mmu05323:Rheumatoid arthritis	18	1,071	1,1E-03	TGFB2, TGFB1, ANGPT1, IL15, TGFB3, CD80, FOS, TNFSF13B, ICAM1, IL6, CTSK, H2-DMB1, ATP6V1B2, ACP5, ATP6V1H, ATP6V0A4, TLR4, H2-AB1	778	87	8941	2,378	3,1E-01	1,7E-02	1,4E-02
mmu01100:Metabolic pathways	173	10,298	1,2E-03	CDA, ALDH1L1, ACAA2, PANK1, SEC1, XYLT1, ACSM5, OGDHL, ENO1, COMT, HNMT, SAT1, ALDH1L2, PPAT, NAMPT, CAR14, ENPP1, CHAC2, HADH, NUDT12, MCCC2, GSTK1, ENTPD1, DGAT2, GPT2, ENTPD5, MCCC1, ALG5, DHDH, ACSL5, CTPS, COX6B2, SDHA, GANC, GCAT, CYP2U1, MTHFD2, ACOT1, SUCLG2, SUCLG1, AMY1, PLPP2, PFKFB1, GCDH, HACD1, NNMT, MAOB, PDE1B, GSTT2, PLD4, ATP5J, GSTT1, ALOX12, PLA2G5, FUT2, MTMR4, COX7A1, SGPL1, MAN2A2, ALDH3B1, LDHD, PLCG2, HYI, ATP6V1H, ATP6V0A4, PRODH, FDFT1, GGT5, MGAM, GSTM1, MTHFR, PYCR1, SYNJ2, ACADSB, DERA, UCK2, ALDH6A1, GGCX, ADI1, PEMT, ALDH18A1, GSTM7, ADA, B4GALT1, ACY1, MOGAT2, AMD1, MTR, GPT, PFAS, AACS, AKR1B8, DHTKD1, NADK2, SPTLC2, ALDH2, NMRK2, AOX3, DBT, PDE4A, ACP5, AOX1, ACADM, UPP1, NANS, IDH3A, PTGIS, CERS6, GPX1, GPX3, PDE4D, PGAM1, SPHK1, PLA2G4B, BCKDHB, PDE4C, AMT, SORD, GPX8, PLA2G4A, GPX7, PGM2L1, INMT, HADHB, ITPKC, HADHA, UGDH, ALDH5A1, PCCA, IVD, ALDH1A2, B3GNT3, ATP6V1B2, PDE5A, NDUFS1, ALDOB, ST6GALNAC4, FBP2, PAFAH1B2, B4GALT5, ALDH9A1, ST6GALNAC6, DDC, ACSS3, HDC, ODC1, NT5C2, TYMS, ADCY7, ADCY6, NT5C, SELENBP1, SELENBP2, ALOX5, MGAT5B, GCNT4, FKTN, DUT, NDUFA5, GCH1, AGL, GATB, ASS1, DHODH, GALC, ALDH4A1, GALNS, NT5C1A, CTH, LPCAT2, GALM, PFKM, PFKP	778	1609	8941	1,236	3,3E-01	1,7E-02	1,4E-02
mmu00760:Nicotinate and nicotinamide metabolism	11	0,655	2,2E-03	NT5C, NADK2, NNMT, NMRK2, NT5C1A, NAMPT, AOX3, AOX1, ENPP1, NT5C2, NUDT12	778	41	8941	3,083	5,0E-01	2,9E-02	2,4E-02
mmu00240:Pyrimidine metabolism	13	0,774	2,7E-03	CDA, ENTPD1, DUT, ENTPD5, CTPS, NT5C2, TYMS, DHODH, NT5C, UCK2, NT5C1A, ENPP1, UPP1	778	56	8941	2,668	5,8E-01	3,4E-02	2,8E-02
mmu04261:Adrenergic signaling in cardiomyocytes	25	1,488	2,9E-03	CAMK2A, ATP2A2, ADRB1, ADRA1B, ADRA1A, ADCY7, ADCY6, GNAI2, PLN, RPS6KA5, CREB3L1, TNNI3, SCN1B, TPM3, CACNA2D2, CACNB1, CACNB2, CREB3, PPP2R2C, BCL2, CALM2, MYH6, ATF4, MYH7, CREB5	778	152	8941	1,890	6,1E-01	3,4E-02	2,8E-02
mmu04020:Calcium signaling pathway	35	2,083	2,9E-03	CHRM2, PTGFR, PDE1B, CAMK2A, ATP2A2, ADRB1, ADRA1B, ADRA1A, ADCY7, CACNA1H, MYLK3, MYLK, MYLK4, FGF6, CYSLTR1, PLN, PHKG1, PDGFC, PLCG2, PTK2B, SPHK1, F2R, AVPR1A, MCOLN2, P2RX7, FGF16, P2RX6, ITPKC, P2RX1, ORAI2, HRC, CASQ1, MET, CALM2, FGF10	778	240	8941	1,676	6,1E-01	3,4E-02	2,8E-02
mmu05144:Malaria	13	0,774	3,1E-03	TGFB2, TGFB1, GYPC, LRP1, TGFB3, THBS2, THBS4, THBS3, ICAM1, IL6, SDC1, TLR4, MET	778	57	8941	2,621	6,3E-01	3,4E-02	2,8E-02
mmu05166:Human T-cell leukemia virus 1 infection	36	2,143	3,1E-03	CDKN1A, H2-K1, SLC2A1, ADCY7, ADCY6, ICAM1, RELB, CCNB2, MYC, H2-DMB1, CREB3L1, TSPO, E2F3, B2M, IL15RA, RANBP1, EGR2, TGFB2, TGFB1, IL15, TGFB3, IL1R2, FOS, TGFBR1, TNFRSF1A, TGFBR2, CREB3, IL6, ESPL1, CANX, LTBR, TLN1, MAP3K14, ATF4, H2-AB1, CREB5	778	250	8941	1,655	6,4E-01	3,4E-02	2,8E-02
mmu04210:Apoptosis	23	1,369	3,1E-03	DFFB, CTSZ, CSF2RB, FOS, ACTB, ACTG1, BBC3, TNFRSF1A, SPTA1, CSF2RB2, TUBA1C, TUBA1B, CASP12, TUBA1A, CTSK, LMNA, IL3RA, BCL2, BAK1, BID, MAP3K14, ATF4, MAP3K5	778	136	8941	1,944	6,4E-01	3,4E-02	2,8E-02
mmu04380:Osteoclast differentiation	22	1,310	3,2E-03	TGFB2, TGFB1, IFNGR1, NCF4, CYBA, TREM2, TNFRSF11B, MITF, FOS, GAB2, TGFBR1, TNFRSF1A, RELB, TGFBR2, FCGR1, TYROBP, FCGR4, CTSK, PLCG2, ACP5, MAP3K14, JUNB	778	128	8941	1,975	6,5E-01	3,4E-02	2,8E-02
mmu00670:One carbon pool by folate	7	0,417	4,3E-03	ALDH1L1, MTHFD2, AMT, MTHFR, MTR, TYMS, ALDH1L2	778	19	8941	4,234	7,5E-01	4,2E-02	3,5E-02
mmu00380:Tryptophan metabolism	12	0,714	4,4E-03	GCDH, HADHA, DDC, MAOB, ALDH2, AOX3, AOX1, CYP1B1, HADH, INMT, ALDH9A1, DHTKD1	778	52	8941	2,652	7,6E-01	4,2E-02	3,5E-02
mmu00071:Fatty acid degradation	12	0,714	4,4E-03	HADHB, GCDH, HADHA, ACAA2, CPT2, ALDH2, CYP2U1, ACSL5, ACADM, HADH, ACADSB, ALDH9A1	778	52	8941	2,652	7,6E-01	4,2E-02	3,5E-02
mmu04072:Phospholipase D signaling pathway	24	1,429	4,7E-03	SHC4, PTGFR, FCER1G, PLA2G4B, SPHK1, F2R, GAB1, LPAR1, RRAS2, PLA2G4A, GAB2, AVPR1A, ADCY7, DNM1, ADCY6, GNA13, CYTH3, CYTH4, RRAS, PDGFC, PLCG2, PTK2B, PLPP2, ARF6	778	149	8941	1,851	7,8E-01	4,3E-02	3,6E-02
mmu04530:Tight junction	26	1,548	4,9E-03	ARPC1B, ACTB, ACTG1, CDC42, TUBA1C, TUBA1B, TUBA1A, MYH11, CGNL1, MAP3K5, ACTR3, VASP, ACTN1, MSN, ARPC4, ACTN4, ARPC5, RUNX1, TJP1, MYL6, PPP2R2C, ARPC2, CTTN, ARPC3, RAPGEF2, HCLS1	778	167	8941	1,789	8,0E-01	4,4E-02	3,7E-02
mmu04728:Dopaminergic synapse	22	1,310	6,1E-03	KCNJ5, KCNJ6, DDC, MAOB, CAMK2A, FOS, GNG12, COMT, GNAI2, ARNTL, GNAO1, CREB3, PPP2R2C, KIF5C, CREB3L1, KIF5B, DRD2, CALM2, SLC18A1, KCNJ3, ATF4, CREB5	778	135	8941	1,873	8,6E-01	5,3E-02	4,4E-02
mmu05140:Leishmaniasis	14	0,833	6,6E-03	MARCKSL1, TGFB2, TGFB1, IFNGR1, TGFB3, NCF4, CYBA, FOS, FCGR1, EEF1A1, FCGR4, H2-DMB1, TLR4, H2-AB1	778	70	8941	2,298	8,8E-01	5,6E-02	4,6E-02
mmu04540:Gap junction	16	0,952	6,7E-03	LPAR1, ADRB1, ADCY7, ADCY6, GNAI2, TJP1, TUBA1C, TUBB2B, TUBA1B, MAPK7, TUBB2A, TUBA1A, TUBB3, PDGFC, CDK1, DRD2	778	86	8941	2,138	8,9E-01	5,6E-02	4,6E-02
mmu05161:Hepatitis B	25	1,488	7,1E-03	CDKN1A, CASP12, MYC, CREB3L1, PTK2B, E2F3, BID, EGR2, TGFB2, EGR3, TGFB1, TGFB3, STAT3, FOS, YWHAZ, TGFBR1, TGFBR2, CREB3, IL6, TRAF3, BCL2, IRF7, TLR4, ATF4, CREB5	778	163	8941	1,763	9,0E-01	5,7E-02	4,8E-02
mmu04919:Thyroid hormone signaling pathway	20	1,190	7,3E-03	THRB, SLC2A1, DIO2, DIO3, ATP2A2, SLC16A10, HIF1A, ACTB, MED12L, ACTG1, RCAN1, PLN, RXRA, TBC1D4, MYC, PLCG2, PFKM, MYH6, PFKP, MYH7	778	120	8941	1,915	9,1E-01	5,7E-02	4,8E-02
mmu04926:Relaxin signaling pathway	21	1,250	7,6E-03	SHC4, TGFB1, MMP2, FOS, GNG12, ADCY7, TGFBR1, ADCY6, TGFBR2, GNAI2, COL1A1, GNAO1, CREB3, COL3A1, COL1A2, COL4A2, COL4A1, CREB3L1, COL4A6, ATF4, CREB5	778	129	8941	1,871	9,2E-01	5,7E-02	4,8E-02
mmu04725:Cholinergic synapse	19	1,131	7,6E-03	CHRM2, ACHE, KCNJ6, KCNJ12, CAMK2A, KCNJ14, FOS, GNG12, ADCY7, ADCY6, GNAI2, GNAO1, CREB3, CREB3L1, BCL2, KCNJ2, KCNJ3, ATF4, CREB5	778	112	8941	1,950	9,2E-01	5,7E-02	4,8E-02
mmu04722:Neurotrophin signaling pathway	20	1,190	8,0E-03	SHC4, SORT1, PRKCD, CAMK2A, ARHGDIG, GAB1, FOXO3, RAP1B, CDC42, MAPK7, RPS6KA5, ARHGDIA, RPS6KA1, NTF3, BCL2, PLCG2, NFKBIE, CALM2, ATF4, MAP3K5	778	121	8941	1,900	9,3E-01	5,9E-02	4,9E-02
mmu04630:JAK-STAT signaling pathway	25	1,488	1,0E-02	CNTFR, CDKN1A, CTF1, FHL1, IL4RA, IL20RB, CSF2RB, CSF2RA, GHR, MYC, AOX3, IL21R, AOX1, IL13RA1, IL15RA, IL15, IFNGR1, STAT3, LIFR, OSMR, CSF2RB2, IL6, IL3RA, BCL2, CRLF2	778	168	8941	1,710	9,6E-01	7,2E-02	6,0E-02

Table S7. KEGG pathway analysis of all DEG in hearts of AAV9-Tfeb but not AAV9-Luc TAC treated mice

Term	Count	%	PValue	Genes	List Total	Pop Hits	Pop Total	Fold Enrichment	Bonferroni	Benjamini	FDR
mmu04935:Growth hormone synthesis, secretion and action	32	1,282	5,0E-06	SHC2, SHC1, IRS1, IRS2, ADCY2, ADCY1, ADCY8, SOCS2, MAPK9, CREB3L2, AKT1, EP300, CACNA1S, MAP2K6, PRKCG, MAP2K3, STAT5A, STAT5B, MAP2K1, MAP2K2, IGFBP3, PRKCA, MAPK14, SSTR3, MTOR, CREB1, ADCY9, GNAS, IGFALS, RAF1, SOS2, BCAR1	1036	116	8941	2,381	0,002	0,002	0,001
mmu04140:Autophagy - animal	35	1,402	2,4E-05	BECN1, IRS1, PIK3R4, IRS2, CAMKK2, PPP2CA, MAPK9, LAMP1, ATG101, CTSL, AKT1, CTSD, CTSB, GABARAPL2, MAP2K1, GABARAPL1, MAP2K2, BAD, DAPK2, DAPK3, ATG9A, ATG10, WIPI1, TSC1, CFLAR, EIF2AK4, MTOR, VMP1, RRAGB, AKT1S1, RRAGD, PRKCQ, SMCR8, RAF1, SQSTM1	1036	142	8941	2,127	0,008	0,004	0,003
mmu04510:Focal adhesion	44	1,762	4,1E-05	ITGB1, SHC2, ROCK1, SHC1, ITGA2B, PDGFA, LAMC1, ARHGAP5, THBS1, ELK1, MYL12A, COMP, VTN, MAPK9, CCND2, KDR, AKT1, PAK6, PIP5K1A, ITGAV, PIP5K1C, ITGB6, PAK3, PAK4, PRKCG, MAP2K1, JUN, LAMB3, EGF, BAD, CAV1, PRKCA, PARVB, VAV1, COL4A4, MYL2, ITGA11, RAPGEF1, COL4A5, RAF1, PPP1R12B, MYL9, SOS2, BCAR1	1036	201	8941	1,889	0,014	0,005	0,004
mmu04910:Insulin signaling pathway	33	1,322	9,2E-05	SHC2, SHC1, IRS1, PRKAG2, IRS2, PYGM, PYGL, PRKCZ, ELK1, PTPRF, HK1, SOCS2, MAPK9, INPP5A, PRKAR2B, MKNK2, FLOT1, AKT1, FLOT2, SH2B2, PTPN1, PRKAB2, MAP2K1, MAP2K2, BAD, TSC1, MTOR, GCK, PPP1R3D, TRIP10, RAPGEF1, RAF1, SOS2	1036	139	8941	2,049	0,030	0,007	0,006
mmu05017:Spinocerebellar ataxia	33	1,322	1,2E-04	BECN1, PSMD11, BEAN1, PSMD14, PSMD13, GTF2B, ATP2A3, PIK3R4, ATP2A1, OMA1, PSMA7, MAPK9, ATXN1, PSMB5, ATG101, PSMB1, ATXN1L, AKT1, GRIA3, PRKCG, TRPC3, NFYA, KCND3, ADRM1, WIPI1, PRKCA, GRIN2C, MTOR, SEM1, AFG3L2, VDAC3, ATXN10, SLC25A5	1036	141	8941	2,020	0,040	0,007	0,006
mmu04926:Relaxin signaling pathway	31	1,241	1,2E-04	SHC2, SHC1, ADCY2, ADCY1, ARRB2, ADCY8, PRKCZ, RXFP1, MAPK9, EDNRB, GNG5, CREB3L2, AKT1, GNG8, NOS1, MAP2K1, JUN, MAP2K2, NOS3, PRKCA, MAPK14, ACTA2, CREB1, ADCY9, COL4A4, GNAS, RLN3, COL4A5, GNB3, RAF1, SOS2	1036	129	8941	2,074	0,041	0,007	0,006
mmu04012:ErbB signaling pathway	22	0,881	4,5E-04	PRKCG, CAMK2B, STAT5A, STAT5B, SHC2, MAP2K1, JUN, MAP2K2, SHC1, EGF, BAD, PRKCA, ELK1, MTOR, MAPK9, ERBB3, AKT1, PAK6, RAF1, PAK3, SOS2, PAK4	1036	84	8941	2,260	0,139	0,021	0,018
mmu03010:Ribosome	37	1,482	6,0E-04	RPL4, RPL10, RPLP1, RPL11, RPLP0, MRPS10, RPL8, MRPL12, MRPL34, RPS15, MRPL20, RPL18A, RPS19, RPL38, RPL37, RPS11, RPL18, RPS10, RPS13, RPL19, RPS7, RPS5, RPL22, MRPL23, RPS26, MRPS9, RPS29, RPL27A, RPS20, RPL27, FAU, RPL26, RPL29, RPL28, UBA52, RPS21, RPS23	1036	179	8941	1,784	0,182	0,025	0,021
mmu05200:Pathways in cancer	88	3,524	8,5E-04	ITGB1, SPI1, ITGA2B, LAMC1, ELK1, FGF2, CCND2, EDNRB, FGF9, HEY1, HEY2, PIM1, AKT1, EP300, ITGAV, ELOB, JAK3, NCOA1, PRKCG, MAP2K1, HSP90AA1, MAP2K2, WNT5B, GSTO1, DAPK2, WNT5A, DAPK3, PRKCA, AXIN2, FH1, DDB2, AR, ADCY9, COL4A4, RARA, COL4A5, RAF1, SOS2, CAMK2B, NOTCH2, CSF1R, HDAC2, RALB, ROCK1, GSTP1, NOTCH4, PTGER3, PDGFA, CXCR4, ADCY2, ADCY1, ADCY8, HSP90B1, RASGRP3, MAPK9, BCL2L11, GNG5, HMOX1, GNG8, HES1, RXRG, STAT5A, NQO1, STAT5B, EGLN3, CDKN2B, JUN, FZD2, LAMB3, TXNRD3, GADD45B, JUP, GADD45A, EGF, BAD, TXNRD1, MLH1, MTOR, AGT, GADD45G, NFKB2, CXCL12, KITL, IL2RA, GNAS, GNB3, FGFR3, FGFR1	1036	543	8941	1,399	0,249	0,032	0,026
mmu04010:MAPK signaling pathway	53	2,123	1,1E-03	PTPRR, RASGRF2, ARRB2, ELK1, DUSP16, FGF2, RPS6KA4, PPP3CB, DUSP10, FGF9, RPS6KA2, KDR, AKT1, MAP3K6, MAP2K3, DUSP4, PRKCG, MAP2K1, MAP2K2, DUSP3, IL1R1, PRKCA, DUSP6, MAPKAPK3, RAF1, SOS2, EPHA2, CSF1R, SRF, PDGFA, RASGRP3, MAPK9, ERBB3, MKNK2, MAP3K20, CACNA1S, MAP2K6, JUN, GADD45B, GADD45A, EGF, NFATC3, HSPA2, MAPK14, GADD45G, NFKB2, NR4A1, KITL, TEK, FGFR3, HSPA1B, HSPA1A, FGFR1	1036	294	8941	1,556	0,305	0,036	0,030
mmu04810:Regulation of actin cytoskeleton	42	1,682	1,3E-03	CYFIP2, ITGB1, CHRM3, ITGAM, ROCK1, ITGA2B, PDGFA, CXCR4, IQGAP2, ACTR3B, FGF2, MYL12A, SCIN, FGF9, CFL2, PAK6, PIP5K1A, ITGAV, NCKAP1L, PIP5K1C, ITGB6, PAK3, MYH10, PAK4, MAP2K1, MAP2K2, EGF, LIMK2, LIMK1, VAV1, CXCL12, MYL2, SPATA13, ITGA11, RAF1, PPP1R12B, ARHGEF7, FGFR3, MYL9, SOS2, BCAR1, FGFR1	1036	220	8941	1,648	0,350	0,036	0,030
mmu04530:Tight junction	34	1,362	1,4E-03	ITGB1, ROCK1, PRKAG2, MYL6B, GATA4, TUBA3B, PRKCZ, ACTR3B, MYL12A, AMOT, PPP2CA, EPB41L4B, MAPK9, PARD6B, MARVELD2, CLDN23, SYNPO, MPDZ, MYH10, MAGI1, PRKAB2, JUN, TUBA4A, TJAP1, CLDN5, DLG1, OCLN, CLDN15, PARD3, MYL2, PPP2R2D, RAB13, MYL9, TUBA8	1036	167	8941	1,757	0,366	0,036	0,030
mmu04360:Axon guidance	36	1,442	1,5E-03	ITGB1, CAMK2B, SEMA5B, NRP1, SEMA7A, ROCK1, SEMA3B, CXCR4, PRKCZ, NTN1, MYL12A, PARD6B, PPP3CB, CFL2, PAK6, PAK3, EPHB4, EPHB3, PAK4, EPHA4, NTNG2, EPHA7, WNT5B, TRPC3, UNC5B, LIMK2, WNT5A, LIMK1, NFATC3, PRKCA, CXCL12, PARD3, PLXNB2, RAF1, MYL9, EPHA2	1036	181	8941	1,717	0,389	0,036	0,030
mmu05166:Human T-cell leukemia virus 1 infection	46	1,842	1,6E-03	H2-T24, H2-T23, NRP1, H2-T22, SPI1, ANAPC15, SRF, H2-DMA, H2-Q4, ADCY2, ADCY1, ADCY8, ELK1, MAPK9, PPP3CB, XPO1, CCND2, PTTG1, CHEK2, CREB3L2, AKT1, EP300, MSX1, JAK3, STAT5A, STAT5B, MAP2K1, JUN, CDKN2B, H2-EB1, RANBP3, MAP2K2, ANAPC7, IL1R1, NFATC3, H2-AA, NFKB2, DLG1, CREB1, ADCY9, IL2RA, CDC16, VDAC3, ATM, SLC25A5, H2-D1	1036	250	8941	1,588	0,415	0,036	0,030
mmu04145:Phagosome	36	1,442	1,6E-03	H2-T24, ITGB1, H2-T23, STX12, SCARB1, H2-T22, ITGAM, NCF1, NCF2, H2-DMA, H2-Q4, TUBA3B, THBS1, CTSS, COMP, LAMP1, CTSL, TUBB1, MRC1, ITGAV, NOS1, SEC61B, ATP6V1F, H2-EB1, ATP6AP1, CD209F, H2-AA, TUBA4A, FCGR3, SEC22B, FCGR2B, H2-D1, ATP6V0E, TLR2, TUBA8, VAMP3	1036	182	8941	1,707	0,419	0,036	0,030
mmu04144:Endocytosis	49	1,962	1,7E-03	H2-T24, ARF3, H2-T23, H2-T22, ARF2, CLTB, AP2A1, ARRB2, CAPZB, CHMP1B, CHMP1A, PSD3, LDLRAP1, CCR5, SH3GL1, PDCD6IP, VPS37B, ZFYVE16, PARD3, CHMP4C, CHMP3, CHMP4B, STAM2, ARF5, H2-D1, H2-Q4, ASAP3, CXCR4, VPS26B, PRKCZ, ACTR3B, SNX4, GRK3, PARD6B, GRK5, AP2S1, PIP5K1A, PIP5K1C, SNX5, ARFGEF1, CAV1, HSPA2, 2610002M06RIK, IL2RA, SMAP2, FGFR3, HSPA1B, SPG21, HSPA1A	1036	272	8941	1,555	0,443	0,037	0,030
mmu00230:Purine metabolism	28	1,121	2,4E-03	PRPS2, PDE1C, PRUNE1, GMPR, ADCY2, ADCY1, ADCY8, NT5E, PNP, GUK1, PGM2, NUDT16, XDH, ADSS, ENTPD2, RRM2, ENTPD6, PDE2A, NME2, APRT, NME1, ADCY9, RRM2B, NME6, PDE3A, ADSSL1, PDE7A, PDE9A	1036	133	8941	1,817	0,553	0,044	0,036
mmu04921:Oxytocin signaling pathway	31	1,241	2,5E-03	CAMK2B, RYR2, ROCK1, PRKAG2, MYL6B, ADCY2, ADCY1, ADCY8, ELK1, PIK3R5, CAMKK2, TRPM2, RGS2, PPP3CB, CACNA1S, PRKCG, KCNJ4, PRKAB2, MAP2K1, JUN, MAP2K2, NOS3, NFATC3, PRKCA, EEF2, ADCY9, CAMK4, GNAS, RAF1, PPP1R12B, MYL9	1036	153	8941	1,749	0,566	0,044	0,036
mmu04022:cGMP-PKG signaling pathway	34	1,362	2,5E-03	OPRD1, ROCK1, IRS1, SRF, ATP2A3, IRS2, ATP2A1, ATP1A2, ADCY2, GATA4, ADCY1, ADCY8, PIK3R5, RGS2, PPP3CB, EDNRB, CREB3L2, AKT1, CACNA1S, GTF2I, MAP2K1, MAP2K2, NOS3, BAD, PDE2A, NFATC3, ATP2B4, CREB1, ADCY9, PDE3A, VDAC3, RAF1, SLC25A5, MYL9	1036	173	8941	1,696	0,566	0,044	0,036
mmu01521:EGFR tyrosine kinase inhibitor resistance	19	0,761	3,4E-03	PRKCG, SHC2, MAP2K1, MAP2K2, SHC1, EGF, BAD, PDGFA, PRKCA, FGF2, MTOR, BCL2L11, ERBB3, KDR, AKT1, RAF1, GAS6, FGFR3, SOS2	1036	79	8941	2,076	0,681	0,053	0,044
mmu04666:Fc gamma R-mediated phagocytosis	21	0,841	3,7E-03	PRKCG, MAP2K1, SYK, NCF1, LIMK2, LIMK1, ASAP3, PRKCA, ACTR3B, VAV1, HCK, SCIN, INPP5D, CFL2, PIP5K1A, AKT1, PIP5K1C, RAF1, PLPP3, FCGR2B, PLPP1	1036	92	8941	1,970	0,714	0,053	0,044
mmu05171:Coronavirus disease - COVID-19	44	1,762	3,8E-03	RPL4, NRP1, C1QA, RPL10, RPLP1, RPL11, RPLP0, RPL8, RPS15, MAPK9, C8G, RPL18A, RPS19, RPL38, RPL37, RPS11, RPL18, RPS10, RPS13, RPL19, PRKCG, JUN, RPS7, SYK, RPS5, RPL22, PRKCA, MAPK14, RPS26, MAS1, OAS2, RPS29, RPL27A, TLR8, RPS20, RPL27, FAU, RPL26, RPL29, RPL28, UBA52, RPS21, TLR2, RPS23	1036	247	8941	1,537	0,723	0,053	0,044
mmu04068:FoxO signaling pathway	27	1,081	3,9E-03	IRS1, SETD7, PRKAG2, IRS2, MAPK9, CCND2, BCL2L11, TNFSF10, AKT1, EP300, HOMER3, GABARAPL2, PRKAB2, MAP2K1, GABARAPL1, CDKN2B, MAP2K2, GADD45B, GADD45A, EGF, FBXO32, MAPK14, KLF2, GADD45G, ATM, RAF1, SOS2	1036	131	8941	1,779	0,734	0,053	0,044
mmu04213:Longevity regulating pathway - multiple species	16	0,641	4,0E-03	PRKAB2, HDAC2, IRS1, PRKAG2, IRS2, ADCY2, ADCY1, HSPA2, ADCY8, MTOR, ADCY9, AKT1S1, AKT1, EIF4EBP2, HSPA1B, HSPA1A	1036	62	8941	2,227	0,736	0,053	0,044
mmu05214:Glioma	18	0,721	4,0E-03	PRKCG, CAMK2B, SHC2, MAP2K1, MAP2K2, GADD45B, SHC1, GADD45A, EGF, PDGFA, PRKCA, MTOR, GADD45G, DDB2, CAMK4, AKT1, RAF1, SOS2	1036	74	8941	2,099	0,738	0,053	0,044
mmu04015:Rap1 signaling pathway	39	1,562	4,5E-03	ITGB1, CSF1R, RALB, ITGAM, ITGA2B, PDGFA, ADCY2, ADCY1, ADCY8, THBS1, PRKCZ, FGF2, RAP1GAP, RASGRP3, PARD6B, FGF9, KDR, AKT1, MAP2K6, PRKCG, MAGI1, MAP2K3, MAP2K1, MAP2K2, EGF, PRKCA, MAPK14, VAV1, ADCY9, KITL, PARD3, GNAS, RAPGEF1, TEK, RAF1, FGFR3, BCAR1, EPHA2, FGFR1	1036	214	8941	1,573	0,780	0,058	0,048
mmu04151:PI3K-Akt signaling pathway	59	2,363	5,0E-03	ITGB1, YWHAB, IRS1, ITGA2B, LAMC1, FGF2, COMP, CCND2, FGF9, CREB3L2, KDR, AKT1, ITGAV, ITGB6, JAK3, MAGI1, MAP2K1, HSP90AA1, MAP2K2, SYK, PPP2R5D, TSC1, PRKCA, CREB1, COL4A4, COL4A5, RAF1, SOS2, EPHA2, TLR2, CSF1R, PHLPP1, PDGFA, THBS1, HSP90B1, PIK3R5, PPP2CA, VTN, BCL2L11, ERBB3, PPP2R3D, GNG5, GNG8, LAMB3, EGF, NOS3, BAD, PPP2R3A, MTOR, NR4A1, KITL, IL2RA, PPP2R2D, ITGA11, GNB3, PKN1, TEK, FGFR3, FGFR1	1036	359	8941	1,418	0,817	0,063	0,052

Table S8. KEGG pathway analysis of all genes that are upregulated in hearts of AAV9-Luc TAC but not AAV9-Tfeb treated mice

Term	Count	%	PValue	Genes	List Tota	Pop Hits	Pop Tota	Fold Enrichmen	Bonferror	Benjamir	FDR
mmu04621:NOD-like receptor signaling pathway	18	4,65	1,72E-06	GSDMD, NLRP1B, CCL12, NLRP1A, CARD9, TNFAIP3, OAS1A, OAS1G, PYCARD, AIM2, NAIP6, IL1B, CASP1, CCL2, NLRP3, PLCB1, MYD88, BIRC3	187	211	8941	4,079	4,5E-04	4,5E-04	4,0E-04
mmu04064:NF-kappa B signaling pathway	12	3,10	1,07E-05	CD40, PRKCB, IL1B, LTA, TNFAIP3, TNFRSF11A, BCL2A1D, PTGS2, BCL2A1B, CARD11, MYD88, BIRC3	187	105	8941	5,464	2,8E-03	1,4E-03	1,2E-03
mmu04110:Cell cycle	12	3,10	5,54E-05	CDC20, CCNA2, CCNB1, CCNE1, TRP53, E2F1, MCM3, E2F2, BUB1B, TTK, CDC25C, BUB1	187	125	8941	4,590	1,4E-02	4,7E-03	4,2E-03
mmu05132:Salmonella infection	17	4,39	7,21E-05	GSDMD, MLKL, RHOG, RHOH, DYNLL1, PYCARD, TUBB6, PAK1, TUBB5, NAIP6, IL1B, CASP1, NLRP3, TLR6, MYD88, S100A10, BIRC3	187	253	8941	3,213	1,9E-02	4,7E-03	4,2E-03
mmu04625:C-type lectin receptor signaling pathway	11	2,84	1,07E-04	PYCARD, CLEC4B1, NRAS, PAK1, CCL22, IL1B, CARD9, CASP1, NLRP3, CALM1, PTGS2	187	112	8941	4,696	2,8E-02	5,6E-03	5,0E-03
mmu05417:Lipid and atherosclerosis	15	3,88	1,59E-04	CCL12, CD40, POU2F2, PYCARD, NRAS, TRP53, IL1B, CASP1, CCL2, NLRP3, TLR6, PLCB1, CALM1, LDLR, MYD88	187	216	8941	3,320	4,1E-02	6,9E-03	6,1E-03
mmu04060:Cytokine-cytokine receptor interaction	17	4,39	3,76E-04	TNFSF18, CCL12, CD40, CCL22, OSM, LIF, INHBA, TNFRSF11A, IL27RA, IL1RL2, IL1B, LTA, CCL2, CD27, CCR7, TNFSF8, IL7R	187	292	8941	2,784	9,4E-02	1,4E-02	1,2E-02
mmu04662:B cell receptor signaling pathway	8	2,07	1,43E-03	NRAS, IFITM1, PIRA2, PRKCB, PIK3AP1, CARD11, CD22, LILRA5	187	81	8941	4,722	3,1E-01	4,7E-02	4,1E-02
mmu04210:Apoptosis	10	2,58	2,08E-03	PARP3, NRAS, TRP53, PRF1, PMAIP1, BIRC5, CTSW, BCL2A1D, BCL2A1B, BIRC3	187	136	8941	3,516	4,2E-01	6,1E-02	5,4E-02
mmu04668:TNF signaling pathway	9	2,33	2,40E-03	CCL12, MLKL, IL1B, LTA, LIF, CCL2, TNFAIP3, PTGS2, BIRC3	187	113	8941	3,808	4,7E-01	6,2E-02	5,5E-02
mmu05163:Human cytomegalovirus infection	14	3,62	2,62E-03	CCL12, PRKCB, H2-M3, PTGS2, EGFR, NRAS, GNGT2, TRP53, IL1B, E2F1, CCL2, E2F2, PLCB1, CALM1	187	256	8941	2,615	5,0E-01	6,2E-02	5,5E-02
mmu05169:Epstein-Barr virus infection	13	3,36	3,16E-03	CD40, H2-M3, TNFAIP3, OAS1A, OAS1G, CCNA2, H2-DMB2, CCNE1, TRP53, E2F1, E2F2, CD247, MYD88	187	231	8941	2,691	5,6E-01	6,5E-02	5,8E-02
mmu05164:Influenza A	11	2,84	3,24E-03	PYCARD, H2-DMB2, CCL12, PRKCB, IL1B, CASP1, CCL2, OAS1A, NLRP3, MYD88, OAS1G	187	173	8941	3,040	5,7E-01	6,5E-02	5,8E-02
mmu05214:Glioma	7	1,81	4,29E-03	NRAS, PRKCB, TRP53, E2F1, E2F2, CALM1, EGFR	187	74	8941	4,523	6,8E-01	7,6E-02	6,7E-02
mmu05200:Pathways in cancer	22	5,68	4,33E-03	PRKCB, WNT9B, PTGS2, EGFR, RASGRP4, CCNA2, NRAS, RAD51, GNGT2, CCNE1, TRP53, GSTA1, STAT4, E2F1, CKS2, PMAIP1, E2F2, BIRC5, IL7R, PLCB1, CALM1, BIRC3	187	543	8941	1,937	6,8E-01	7,6E-02	6,7E-02
mmu05133:Pertussis	7	1,81	5,22E-03	PYCARD, IL1B, CASP1, NLRP3, CALM1, MYD88, C2	187	77	8941	4,347	7,5E-01	8,5E-02	7,6E-02
mmu05144:Malaria	6	1,55	6,45E-03	CD40, CCL12, IL1B, CCL2, ACKR1, MYD88	187	57	8941	5,033	8,2E-01	9,9E-02	8,8E-02
mmu05161:Hepatitis B	10	2,58	6,90E-03	CCNA2, NRAS, CCNE1, PRKCB, TRP53, STAT4, E2F1, E2F2, BIRC5, MYD88	187	163	8941	2,933	8,4E-01	1,0E-01	8,9E-02
mmu04540:Gap junction	7	1,81	8,88E-03	NRAS, TUBB6, TUBB5, PRKCB, PLCB1, EGFR, PRKG1	187	86	8941	3,892	9,0E-01	1,2E-01	1,1E-01
mmu05219:Bladder cancer	5	1,29	1,01E-02	NRAS, TRP53, E2F1, E2F2, EGFR	187	41	8941	5,831	9,3E-01	1,3E-01	1,2E-01
mmu05206:MicroRNAs in cancer	14	3,62	1,06E-02	PRKCB, CDCA5, KIF23, CDC25C, PTGS2, EGFR, NRAS, MARCKS, SOCS1, CCNE1, TRP53, STMN1, E2F1, E2F2	187	303	8941	2,209	9,4E-01	1,3E-01	1,2E-01
mmu05170:Human immunodeficiency virus 1 infection	12	3,10	1,15E-02	BST2, NRAS, CCNB1, PAK1, GNGT2, TRIM30D, PRKCB, H2-M3, CD247, CDC25C, CALM1, MYD88	187	240	8941	2,391	9,5E-01	1,4E-01	1,2E-01
mmu05222:Small cell lung cancer	7	1,81	1,28E-02	CCNE1, TRP53, E2F1, CKS2, E2F2, PTGS2, BIRC3	187	93	8941	3,599	9,7E-01	1,5E-01	1,3E-01
mmu05171:Coronavirus disease - COVID-19	12	3,10	1,41E-02	CCL12, PRKCB, IL1B, C5AR1, CASP1, CCL2, OAS1A, NLRP3, EGFR, MYD88, C2, OAS1G	187	247	8941	2,323	9,8E-01	1,5E-01	1,4E-01
mmu04218:Cellular senescence	10	2,58	1,46E-02	CCNA2, NRAS, CCNB1, CCNE1, TRP53, H2-M3, E2F1, E2F2, CALM1, FOXM1	187	184	8941	2,599	9,8E-01	1,5E-01	1,4E-01
mmu05166:Human T-cell leukemia virus 1 infection	12	3,10	1,52E-02	CDC20, CCNA2, H2-DMB2, NRAS, CD40, CCNE1, TRP53, H2-M3, LTA, E2F1, E2F2, BUB1B	187	250	8941	2,295	9,8E-01	1,5E-01	1,4E-01
mmu05223:Non-small cell lung cancer	6	1,55	1,68E-02	NRAS, PRKCB, TRP53, E2F1, E2F2, EGFR	187	72	8941	3,984	9,9E-01	1,6E-01	1,4E-01
mmu05142:Chagas disease	7	1,81	2,03E-02	CCL12, IL1B, CCL2, CD247, TLR6, PLCB1, MYD88	187	103	8941	3,249	1,0E+00	1,9E-01	1,7E-01
mmu05135:Yersinia infection	8	2,07	2,16E-02	PYCARD, CCL12, IL1B, RHOG, CASP1, CCL2, NLRP3, MYD88	187	134	8941	2,854	1,0E+00	2,0E-01	1,7E-01
mmu01524:Platinum drug resistance	6	1,55	2,53E-02	TOP2A, TRP53, GSTA1, PMAIP1, BIRC5, BIRC3	187	80	8941	3,586	1,0E+00	2,2E-01	2,0E-01
mmu05145:Toxoplasmosis	7	1,81	2,70E-02	H2-DMB2, CD40, SOCS1, TLR11, LDLR, MYD88, BIRC3	187	110	8941	3,043	1,0E+00	2,3E-01	2,0E-01
mmu05168:Herpes simplex virus 1 infection	17	4,39	2,95E-02	CCL12, ALYREF, CARD9, H2-M3, ZFP954, OAS1A, PILRA, POU2F2, OAS1G, BST2, H2-DMB2, TRP53, IL1B, LTA, CCL2, MYD88, BIRC3	187	459	8941	1,771	1,0E+00	2,4E-01	2,1E-01
mmu04217:Necroptosis	9	2,33	3,05E-02	PYCARD, ZBP1, MLKL, IL1B, STAT4, CASP1, TNFAIP3, NLRP3, BIRC3	187	176	8941	2,445	1,0E+00	2,4E-01	2,1E-01
mmu04970:Salivary secretion	6	1,55	3,32E-02	PRKCB, LYZ1, ATP1A3, CALM1, PLCB1, PRKG1	187	86	8941	3,336	1,0E+00	2,6E-01	2,3E-01
mmu05323:Rheumatoid arthritis	6	1,55	3,47E-02	ATP6V1A, H2-DMB2, CCL12, IL1B, CCL2, TNFRSF11A	187	87	8941	3,297	1,0E+00	2,6E-01	2,3E-01
mmu05235:PD-L1 expression and PD-1 checkpoint pathway in cancer	6	1,55	3,62E-02	NRAS, BATF3, CD247, EGFR, MYD88, BATF	187	88	8941	3,260	1,0E+00	2,6E-01	2,3E-01
mmu05134:Legionellosis	5	1,29	3,78E-02	PYCARD, NAIP6, IL1B, CASP1, MYD88	187	61	8941	3,919	1,0E+00	2,7E-01	2,4E-01
mmu04114:Oocyte meiosis	7	1,81	4,03E-02	CDC20, CCNB1, CCNE1, CDC25C, CALM1, BUB1, AURKA	187	121	8941	2,766	1,0E+00	2,7E-01	2,4E-01
mmu04657:IL-17 signaling pathway	6	1,55	4,09E-02	CCL12, IL1B, CCL2, TNFAIP3, PTGS2, S100A9	187	91	8941	3,152	1,0E+00	2,7E-01	2,4E-01
mmu04623:Cytosolic DNA-sensing pathway	5	1,29	4,18E-02	PYCARD, ZBP1, AIM2, IL1B, CASP1	187	63	8941	3,795	1,0E+00	2,7E-01	2,4E-01
mmu04914:Progesterone-mediated oocyte maturation	6	1,55	4,25E-02	CCNA2, CCNB1, KIF22, CDC25C, BUB1, AURKA	187	92	8941	3,118	1,0E+00	2,7E-01	2,4E-01
mmu05143:African trypanosomiasis	4	1,03	4,68E-02	PRKCB, IL1B, PLCB1, MYD88	187	39	8941	4,904	1,0E+00	2,9E-01	2,5E-01
mmu04062:Chemokine signaling pathway	9	2,33	4,70E-02	NRAS, CCL12, PAK1, GNGT2, CCL22, PRKCB, CCL2, CCR7, PLCB1	187	192	8941	2,241	1,0E+00	2,9E-01	2,5E-01

Table S9. KEGG pathway analysis of all genes that are upregulated in hearts of AAV9-Luc TAC and AAV9-Tfeb treated mice

Term	Count	%	PValue	Genes	List Tota	Pop Hits	Pop Tota	Fold Enrichmen	Bonferror	Benjamin	FDR
mmu05132:Salmonella infection	40	4,16	1,38E-09	RAB5C, ARPC1B, ACTB, ACTG1, CYTH3, CDC42, TUBA1C, TUBA1B, TUBA1A, CYTH4, RRAS, TUBB3, KIF5C, MYC, KIF5B, CASP4, BAK1, CD14, FLNC, ACTR3, ANXA2, AHNAK2, ARPC4, FOS, ARPC5, TNFRSF1A, DYNC1LI1, IL6, TUBB2B, TUBB2A, ARPC2, ARPC3, RHOJ, BCL2, ELMO1, PFN1, RAB7B, TLR4, ARL8A, ARF6	477	253	8941	2,964	4,1E-07	3,5E-07	2,8E-07
mmu05205:Proteoglycans in cancer	35	3,64	2,38E-09	CD63, CDKN1A, ITGB5, SDC4, HIF1A, ACTB, ACTG1, CDC42, RRAS, MYC, PLCG2, FLNC, FZD1, TGFB2, TGFB1, CAV3, LUM, MMP2, STAT3, PLAUR, FN1, MSN, RRAS2, IGF1, DCN, COL1A1, COL1A2, CTTN, SDC1, HCLS1, ITGA5, TLR4, MET, CD44, HBEGF	477	205	8941	3,200	7,1E-07	3,5E-07	2,8E-07
mmu04512:ECM-receptor interaction	22	2,29	4,10E-09	ITGB5, SDC4, TNC, FN1, LAMC2, HMMR, THBS4, THBS3, COL1A1, COL1A2, COL4A2, COL4A1, COL6A2, COL6A1, SPP1, TNR, SDC1, COL9A2, ITGA5, FREM1, CD44, ITGA9	477	88	8941	4,686	1,2E-06	4,0E-07	3,1E-07
mmu04510:Focal adhesion	34	3,54	5,37E-09	SHC4, ITGB5, TNC, LAMC2, THBS4, ACTB, THBS3, ACTG1, RAP1B, CDC42, PDGFC, SPP1, TNR, FLNC, VASP, CAV3, ACTN1, FN1, IGF1, ACTN4, COL1A1, DIAPH1, COL1A2, COL4A2, COL4A1, COL6A2, COL6A1, ZYX, BCL2, ITGA5, COL9A2, TLN1, MET, ITGA9	477	201	8941	3,171	1,6E-06	4,0E-07	3,1E-07
mmu04145:Phagosome	31	3,23	2,47E-08	COLEC12, RAB5C, TFRC, ITGB5, NCF4, H2-K1, CORO1A, THBS4, ACTB, THBS3, ACTG1, MRC2, TUBA1C, TUBA1B, TUBA1A, TUBB3, H2-DMB1, ATP6V1H, ATP6V0A4, CD14, CYBA, FCGR1, DYNC1LI1, TUBB2B, TUBB2A, FCGR4, ATP6V1B2, ITGA5, RAB7B, TLR4, H2-AB1	477	182	8941	3,193	7,4E-06	1,5E-06	1,1E-06
mmu04933:AGE-RAGE signaling pathway in diabetic complications	21	2,19	2,69E-07	TGFB2, TGFB1, TGFB3, MMP2, PRKCD, SERPINE1, STAT3, FN1, TGFBR1, TGFBR2, ICAM1, COL1A1, CDC42, DIAPH1, IL6, COL3A1, COL1A2, COL4A2, COL4A1, BCL2, PLCG2	477	101	8941	3,897	8,0E-05	1,3E-05	1,0E-05
mmu05100:Bacterial invasion of epithelial cells	18	1,87	3,43E-07	ACTR3, SHC4, CAV3, ARPC1B, FN1, ARPC4, ARPC5, ACTB, DNM1, ACTG1, CDC42, ARPC2, CTTN, ARPC3, ELMO1, HCLS1, ITGA5, MET	477	76	8941	4,439	1,0E-04	1,5E-05	1,1E-05
mmu04974:Protein digestion and absorption	21	2,19	8,33E-07	COL15A1, COL16A1, COL14A1, ELN, COL12A1, SLC3A2, PRCP, COL1A1, COL3A1, COL1A2, COL4A2, COL5A1, COL4A1, COL6A2, COL5A3, COL5A2, COL6A1, COL8A2, COL8A1, COL9A2, SLC38A2	477	108	8941	3,645	2,5E-04	3,1E-05	2,4E-05
mmu04666:Fc gamma R-mediated phagocytosis	19	1,98	1,28E-06	ACTR3, VASP, MARCKSL1, SPHK1, ARPC1B, PRKCD, PLA2G4A, ARPC4, GAB2, ARPC5, FCGR1, CDC42, ARPC2, FCGR4, ARPC3, CFL1, PLCG2, PLPP2, ARF6	477	92	8941	3,871	3,8E-04	4,2E-05	3,3E-05
mmu04810:Regulation of actin cytoskeleton	31	3,23	1,73E-06	ITGB5, ARPC1B, LPAR1, IQGAP3, ACTB, ACTG1, GNA13, CDC42, FGF6, RRAS, CFL1, PDGFC, ACTR3, ACTN1, F2R, FN1, MSN, RRAS2, ARPC4, ACTN4, ARPC5, BAIAP2, GNG12, ENAH, DIAPH1, DIAPH3, ARPC2, ARPC3, ITGA5, PFN1, ITGA9	477	220	8941	2,641	5,2E-04	5,2E-05	4,0E-05
mmu05146:Amoebiasis	20	2,08	3,03E-06	TGFB2, TGFB1, RAB5C, TGFB3, ACTN1, IL1R2, FN1, HSPB1, LAMC2, ACTN4, COL1A1, IL6, COL3A1, COL1A2, COL4A2, SERPINB6B, COL4A1, CD14, RAB7B, TLR4	477	107	8941	3,504	9,0E-04	8,2E-05	6,4E-05
mmu04060:Cytokine-cytokine receptor interaction	36	3,75	4,94E-06	CX3CR1, IL4RA, IL20RB, TNFRSF11B, CSF2RB, CXCR6, CSF2RA, CX3CL1, TNFSF13B, CXCL16, CCL8, IL21R, RELT, IL13RA1, TGFB2, XCR1, TGFB1, TNFRSF12A, GDF15, IFNGR1, TGFB3, IL1R2, INHBB, GDF6, OSMR, TNFRSF1B, TGFBR1, IL17RA, TNFRSF1A, TGFBR2, CSF2RB2, IL6, IL3RA, ACKR4, LTBR, CRLF2	477	292	8941	2,311	1,5E-03	1,1E-04	8,9E-05
mmu05161:Hepatitis B	25	2,60	5,01E-06	CDKN1A, CASP12, MYC, CREB3L1, PTK2B, E2F3, BID, EGR2, TGFB2, EGR3, TGFB1, TGFB3, STAT3, FOS, YWHAZ, TGFBR1, TGFBR2, CREB3, IL6, TRAF3, BCL2, IRF7, TLR4, ATF4, CREB5	477	163	8941	2,875	1,5E-03	1,1E-04	8,9E-05
mmu05166:Human T-cell leukemia virus 1 infection	32	3,33	8,68E-06	CDKN1A, H2-K1, SLC2A1, ADCY7, ICAM1, RELB, CCNB2, MYC, H2-DMB1, CREB3L1, TSPO, E2F3, B2M, RANBP1, EGR2, TGFB2, TGFB1, TGFB3, IL1R2, FOS, TGFBR1, TNFRSF1A, TGFBR2, CREB3, IL6, ESPL1, LTBR, TLN1, MAP3K14, ATF4, H2-AB1, CREB5	477	250	8941	2,399	2,6E-03	1,8E-04	1,4E-04
mmu04380:Osteoclast differentiation	21	2,19	1,26E-05	TGFB2, TGFB1, IFNGR1, NCF4, CYBA, TREM2, TNFRSF11B, FOS, GAB2, TGFBR1, TNFRSF1A, RELB, TGFBR2, FCGR1, TYROBP, FCGR4, CTSK, PLCG2, ACP5, MAP3K14, JUNB	477	128	8941	3,075	3,7E-03	2,5E-04	1,9E-04
mmu04151:PI3K-Akt signaling pathway	40	4,16	1,52E-05	CDKN1A, ITGB5, FLT3, LPAR1, TNC, IL4RA, LAMC2, THBS4, THBS3, FGF6, MYC, CREB3L1, PDGFC, SPP1, EIF4EBP1, TNR, F2R, FN1, IGF1, GNG12, OSMR, YWHAZ, EREG, COL1A1, CREB3, IL6, COL1A2, COL4A2, COL4A1, COL6A2, COL6A1, IL3RA, BCL2, ITGA5, COL9A2, TLR4, MET, ATF4, ITGA9, CREB5	477	359	8941	2,088	4,5E-03	2,8E-04	2,2E-04
mmu05171:Coronavirus disease - COVID-19	31	3,23	1,83E-05	C1QB, RPL3, RPL31, RPL12, RPL34, RPL36A, RPS27L, RPL10A, C4B, RPS16, PLCG2, RPL13, RPL35, RPS27A, RPL17, RPL39, RPS12, RPL41, ACE, MX1, STAT3, FOS, TNFRSF1A, IL6, RPS28, TRAF3, TLR7, MASP1, TLR4, HBEGF, C1QC	477	247	8941	2,353	5,5E-03	3,2E-04	2,5E-04
mmu05152:Tuberculosis	25	2,60	2,77E-05	RAB5C, LSP1, CORO1A, MRC2, H2-DMB1, ATP6V1H, ATP6V0A4, LBP, CD14, BID, PLK3, TGFB2, TGFB1, FCER1G, IFNGR1, TGFB3, SPHK1, TNFRSF1A, FCGR1, IL6, FCGR4, BCL2, TLR4, CALM2, H2-AB1	477	180	8941	2,603	8,2E-03	4,6E-04	3,6E-04
mmu05323:Rheumatoid arthritis	16	1,66	4,75E-05	TGFB2, TGFB1, TGFB3, CD80, FOS, TNFSF13B, ICAM1, IL6, CTSK, H2-DMB1, ATP6V1B2, ACP5, ATP6V1H, ATP6V0A4, TLR4, H2-AB1	477	87	8941	3,447	1,4E-02	7,5E-04	5,8E-04
mmu05140:Leishmaniasis	14	1,46	6,83E-05	MARCKSL1, TGFB2, TGFB1, IFNGR1, TGFB3, NCF4, CYBA, FOS, FCGR1, EEF1A1, FCGR4, H2-DMB1, TLR4, H2-AB1	477	70	8941	3,749	2,0E-02	1,0E-03	7,9E-04
mmu05410:Hypertrophic cardiomyopathy	16	1,66	8,14E-05	TGFB2, TGFB1, ACE, ITGB5, TGFB3, TPM3, IGF1, ACTB, ACTG1, CACNB1, IL6, DES, LMNA, ITGA5, ITGA9, MYH7	477	91	8941	3,296	2,4E-02	1,1E-03	8,6E-04
mmu05135:Yersinia infection	20	2,08	8,18E-05	ACTR3, ARPC1B, FN1, ARPC4, FOS, ARPC5, BAIAP2, ACTB, ACTG1, CDC42, IL6, ARPC2, ARPC3, RPS6KA1, ELMO1, PTK2B, ITGA5, TLR4, ARF6, SKAP2	477	134	8941	2,798	2,4E-02	1,1E-03	8,6E-04
mmu04210:Apoptosis	20	2,08	1,00E-04	CTSZ, CSF2RB, FOS, ACTB, ACTG1, BBC3, TNFRSF1A, CSF2RB2, TUBA1C, TUBA1B, CASP12, TUBA1A, CTSK, LMNA, IL3RA, BCL2, BAK1, BID, MAP3K14, ATF4	477	136	8941	2,757	2,9E-02	1,3E-03	9,9E-04
mmu04066:HIF-1 signaling pathway	18	1,87	1,02E-04	CDKN1A, TFRC, IFNGR1, SERPINE1, STAT3, SLC2A1, IGF1, ENO1, HIF1A, IL6, NPPA, BCL2, EIF4EBP1, PLCG2, TIMP1, LTBR, TLR4, PFKP	477	114	8941	2,960	3,0E-02	1,3E-03	9,9E-04
mmu04926:Relaxin signaling pathway	19	1,98	1,54E-04	SHC4, TGFB1, MMP2, FOS, GNG12, ADCY7, TGFBR1, TGFBR2, GNAI2, COL1A1, GNAO1, CREB3, COL3A1, COL1A2, COL4A2, COL4A1, CREB3L1, ATF4, CREB5	477	129	8941	2,761	4,5E-02	1,8E-03	1,4E-03
mmu04010:MAPK signaling pathway	32	3,33	2,00E-04	FLT3, HSPB1, RELB, RAP1B, CDC42, FGF6, MAPK7, RRAS, MYC, RPS6KA1, PDGFC, CD14, FLNC, DUSP5, DUSP2, TGFB2, TGFB1, TGFB3, PLA2G4A, RRAS2, IGF1, FOS, GNG12, TGFBR1, TNFRSF1A, EREG, TGFBR2, CACNB1, PTPN7, MET, MAP3K14, ATF4	477	294	8941	2,040	5,8E-02	2,3E-03	1,8E-03
mmu05200:Pathways in cancer	49	5,10	3,13E-04	CDKN1A, FLT3, SLC2A1, LAMC2, BBC3, FGF6, MYC, RASSF5, IL13RA1, TPM3, IFNGR1, MMP2, F2R, FOS, TGFBR1, TGFBR2, RUNX1, CSF2RB2, COL4A2, TRAF3, COL4A1, IL3RA, MET, PTGER4, CEBPA, LPAR1, IL4RA, CSF2RB, HIF1A, CSF2RA, ADCY7, GNAI2, GNA13, CDC42, PLCG2, E2F3, BAK1, BID, FZD1, TGFB2, TGFB1, TGFB3, STAT3, FN1, IGF1, GNG12, IL6, BCL2, CALM2	477	543	8941	1,691	8,9E-02	3,5E-03	2,7E-03
mmu05142:Chagas disease	16	1,66	3,37E-04	C1QB, TGFB2, TGFB1, ACE, IFNGR1, TGFB3, SERPINE1, FOS, TGFBR1, TNFRSF1A, TGFBR2, GNAI2, GNAO1, IL6, TLR4, C1QC	477	103	8941	2,912	9,5E-02	3,6E-03	2,8E-03
mmu05414:Dilated cardiomyopathy	15	1,56	4,17E-04	TGFB2, TGFB1, ITGB5, TGFB3, TPM3, IGF1, ADCY7, ACTB, ACTG1, CACNB1, DES, LMNA, ITGA5, ITGA9, MYH7	477	94	8941	2,991	1,2E-01	4,1E-03	3,2E-03

Table S10. KEGG pathway analysis of all genes that are upregulated in hearts of AAV9-Tfeb but not AAV9-Luc TAC treated mice

Term	Count	%	PValue	Genes	List Tota	Pop Hits	Pop Tota	Fold Enrichmen	Bonferror	Benjamir	FDR
mmu03010:Ribosome	35	2,58	1,3E-08	RPL4, RPL10, RPLP1, RPL11, RPLP0, MRPS10, RPL8, MRPL12, MRPL34, RPS15, MRPL20, RPL18A, RPS19, RPL38, RPL37, RPS11, RPL18, RPS10, RPS13, RPL19, RPS7, RPS5, RPL22, RPS26, RPS29, RPL27A, RPS20, RPL27, FAU, RPL26, RPL29, RPL28, UBA52, RPS21, RPS23	587	179	8941	2,978	4,2E-06	4,2E-06	3,8E-06
mmu05171:Coronavirus disease - COVID-19	38	2,80	1,7E-06	RPL4, C1QA, RPL10, RPLP1, RPL11, RPLP0, RPL8, RPS15, RPL18A, RPS19, RPL38, RPL37, RPS11, RPL18, RPS10, RPS13, RPL19, PRKCG, JUN, RPS7, SYK, RPS5, RPL22, PRKCA, RPS26, RPS29, RPL27A, TLR8, RPS20, RPL27, FAU, RPL26, RPL29, RPL28, UBA52, RPS21, TLR2, RPS23	587	247	8941	2,343	5,4E-04	2,4E-04	2,2E-04
mmu04145:Phagosome	31	2,28	2,2E-06	ITGB1, H2-T23, STX12, SCARB1, H2-T22, ITGAM, NCF1, NCF2, H2-DMA, H2-Q4, TUBA3B, THBS1, CTSS, COMP, LAMP1, CTSL, MRC1, ITGAV, NOS1, SEC61B, ATP6V1F, H2-EB1, ATP6AP1, H2-AA, FCGR3, SEC22B, FCGR2B, H2-D1, ATP6V0E, TLR2, VAMP3	587	182	8941	2,594	7,2E-04	2,4E-04	2,2E-04
mmu04144:Endocytosis	37	2,72	3,8E-05	ARF3, H2-T23, H2-T22, ARF2, CLTB, H2-Q4, CXCR4, AP2A1, ARRB2, SNX4, GRK3, PARD6B, GRK5, CAPZB, CHMP1B, CHMP1A, AP2S1, PIP5K1A, PIP5K1C, CCR5, LDLRAP1, SNX5, SH3GL1, PDCD6IP, VPS37B, HSPA2, ZFYVE16, CHMP4C, CHMP3, CHMP4B, STAM2, FGFR3, ARF5, HSPA1B, H2-D1, SPG21, HSPA1A	587	272	8941	2,072	1,2E-02	3,1E-03	2,8E-03
mmu04666:Fc gamma R-mediated phagocytosis	18	1,32	8,3E-05	PRKCG, MAP2K1, SYK, NCF1, LIMK2, LIMK1, PRKCA, VAV1, HCK, SCIN, INPP5D, CFL2, PIP5K1A, AKT1, PIP5K1C, PLPP3, FCGR2B, PLPP1	587	92	8941	2,980	2,7E-02	5,4E-03	4,9E-03
mmu05017:Spinocerebellar ataxia	23	1,69	1,2E-04	PRKCG, BECN1, PSMD11, BEAN1, PSMD14, PSMD13, GTF2B, ADRM1, PRKCA, WIPI1, GRIN2C, PSMA7, MTOR, SEM1, AFG3L2, PSMB5, ATG101, PSMB1, ATXN1L, AKT1, ATXN10, SLC25A5, GRIA3	587	141	8941	2,485	3,7E-02	6,0E-03	5,5E-03
mmu04140:Autophagy - animal	23	1,69	1,3E-04	GABARAPL2, BECN1, MAP2K1, GABARAPL1, MAP2K2, BAD, DAPK2, DAPK3, IRS2, WIPI1, EIF2AK4, MTOR, CAMKK2, VMP1, PPP2CA, LAMP1, CTSL, ATG101, AKT1S1, AKT1, CTSD, SQSTM1, CTSB	587	142	8941	2,467	4,1E-02	6,0E-03	5,5E-03
mmu04910:Insulin signaling pathway	22	1,62	2,6E-04	PTPN1, PRKAB2, SHC2, MAP2K1, MAP2K2, SHC1, BAD, PRKAG2, IRS2, PYGL, PTPRF, MTOR, GCK, HK1, SOCS2, INPP5A, MKNK2, RAPGEF1, FLOT1, AKT1, FLOT2, SH2B2	587	139	8941	2,411	8,2E-02	1,1E-02	9,7E-03
mmu04510:Focal adhesion	27	1,99	6,4E-04	ITGB1, SHC2, SHC1, LAMC1, THBS1, MYL12A, COMP, CCND2, AKT1, PAK6, PIP5K1A, ITGAV, PIP5K1C, PAK3, PAK4, PRKCG, MAP2K1, JUN, BAD, PRKCA, PARVB, VAV1, COL4A4, ITGA11, RAPGEF1, COL4A5, BCAR1	587	201	8941	2,046	1,9E-01	2,1E-02	1,9E-02
mmu04612:Antigen processing and presentation	16	1,18	6,9E-04	H2-T23, CD74, H2-EB1, H2-T22, HSP90AA1, H2-DMA, H2-Q4, RFXANK, HSPA2, H2-AA, CTSS, CTSL, H2-D1, HSPA1B, CTSB, HSPA1A	587	90	8941	2,708	2,0E-01	2,1E-02	1,9E-02
mmu04926:Relaxin signaling pathway	20	1,47	7,0E-04	SHC2, MAP2K1, JUN, MAP2K2, SHC1, NOS3, PRKCA, ADCY2, ARRB2, EDNRB, GNG5, COL4A4, CREB3L2, GNAS, AKT1, GNB3, RLN3, GNG8, COL4A5, NOS1	587	129	8941	2,362	2,0E-01	2,1E-02	1,9E-02
mmu04142:Lysosome	20	1,47	1,2E-03	ATP6AP1, HEXB, FUCA1, SLC11A1, GBA, CLTB, LAPTM5, ABCB9, CTSS, SUMF1, GM2A, LAMP1, CTSL, HYAL2, SMPD1, DMXL2, AP1S3, CTSD, CTSB, AP1M1	587	135	8941	2,257	3,3E-01	3,3E-02	3,0E-02
mmu01250:Biosynthesis of nucleotide sugars	9	0,66	2,7E-03	GPI1, UGP2, PMM1, MPI, UAP1L1, PGM2, GCK, GALK1, HK1	587	38	8941	3,608	5,9E-01	6,8E-02	6,1E-02
mmu04530:Tight junction	22	1,62	2,9E-03	MAGI1, ITGB1, PRKAB2, JUN, PRKAG2, MYL6B, GATA4, TUBA3B, MYL12A, TJAP1, AMOT, PPP2CA, CLDN5, PARD6B, OCLN, CLDN15, MARVELD2, CLDN23, RAB13, PPP2R2D, SYNPO, MYH10	587	167	8941	2,007	6,1E-01	6,8E-02	6,1E-02
mmu04935:Growth hormone synthesis, secretion and action	17	1,25	3,6E-03	PRKCG, MAP2K3, SHC2, MAP2K1, MAP2K2, SHC1, IGFBP3, IRS2, PRKCA, ADCY2, MTOR, SOCS2, CREB3L2, GNAS, AKT1, IGFALS, BCAR1	587	116	8941	2,232	6,9E-01	7,7E-02	7,0E-02
mmu05170:Human immunodeficiency virus 1 infection	28	2,06	4,0E-03	H2-T23, H2-T22, H2-Q4, CXCR4, GNG5, CFL2, AKT1, PAK6, GNG8, ELOB, AP1S3, CCR5, PAK3, AP1M1, PAK4, PRKCG, MAP2K3, MAP2K1, JUN, MAP2K2, BAD, LIMK2, LIMK1, PRKCA, MTOR, GNB3, H2-D1, TLR2	587	240	8941	1,777	7,3E-01	8,2E-02	7,4E-02
mmu00520:Amino sugar and nucleotide sugar metabolism	10	0,74	5,3E-03	GPI1, UGP2, PMM1, HEXB, MPI, UAP1L1, PGM2, GCK, GALK1, HK1	587	51	8941	2,987	8,2E-01	9,9E-02	9,0E-02
mmu05221:Acute myeloid leukemia	12	0,88	5,5E-03	CSF1R, MAP2K1, SPI1, ITGAM, MAP2K2, JUP, BAD, PIM1, RARA, AKT1, DUSP6, MTOR	587	70	8941	2,611	8,3E-01	9,9E-02	9,0E-02
mmu04062:Chemokine signaling pathway	23	1,69	7,2E-03	CCR1, SHC2, MAP2K1, NCF1, SHC1, BAD, CXCR4, ADCY2, ARRB2, VAV1, PIK3R5, PREX1, HCK, GRK3, GNG5, GRK5, AKT1, GNB3, GNG8, CCR5, JAK3, BCAR1, CCR2	587	192	8941	1,825	9,1E-01	1,2E-01	1,1E-01
mmu01100:Metabolic pathways	129	9,49	8,0E-03	CHPF, CERK, GALNT16, NDUFA11, GALNT18, EHMT2, MPI, ABAT, COX6A1, CNDP2, PNP, FPGS, NUDT16, SEPHS2, GLUL, ENTPD2, DGAT1, MIF, PGD, INPP4B, DPM3, PLPP3, PLPP1, PDE9A, CDS1, MTMR2, PDE1C, SHMT1, GMPR, PLA2G7, PLD3, PTDSS1, INPP5A, RDH11, INPP5D, UAP1L1, ESD, ATP5D, SMYD2, INPP5J, PGK1, HMOX1, PIP5K1A, SMYD1, ST8SIA5, ASL, B4GALNT1, PIP5K1C, HMOX2, ATP6V1F, PMM1, AZIN2, GRHPR, ALPL, GALK1, B4GALT2, PIGT, HEXB, SETD7, PYGL, HK1, GPI1, GYG, GUK1, SMPD1, HYAL2, PGM2, PGLS, NOS1, A4GALT, COX8A, VKORC1, ELOVL1, TPI1, GPX4, ATP6AP1, GSTO1, CHPF2, PGAM2, NME2, AKR1A1, POMGNT1, APRT, NME1, ALDH1A3, BDH1, DAD1, ALDH1A1, ADSSL1, PIGK, BLVRB, ACOX3, HAGH, BCAT1, GAPDH, BLVRA, CERS2, ATP6V0E, AHCY, GBA, PRUNE1, ADCY2, HSD17B7, AGPAT2, SRM, CKMT1, NT5E, UGP2, MTHFD1L, LAO1, MAN1C1, MGAT1, CKB, CDIPT, ADSS, NQO1, TIGAR, RRM2, GK, NOS3, SCD4, HSD3B1, GCK, DHRS9, MGAT4A, SARDH, PLCD3, LPIN2, LPIN3	587	1609	8941	1,221	9,3E-01	1,3E-01	1,1E-01
mmu04012:ErbB signaling pathway	13	0,96	8,3E-03	PRKCG, SHC2, MAP2K1, JUN, MAP2K2, SHC1, BAD, PRKCA, MTOR, AKT1, PAK6, PAK3, PAK4	587	84	8941	2,357	9,3E-01	1,3E-01	1,1E-01
mmu04072:Phospholipase D signaling pathway	19	1,40	8,5E-03	SHC2, MAP2K1, ARF2, RALB, MAP2K2, SYK, SHC1, PRKCA, ADCY2, AGPAT2, MTOR, AGT, PIK3R5, GNAS, PIP5K1A, AKT1, PIP5K1C, PLPP3, PLPP1	587	149	8941	1,942	9,4E-01	1,3E-01	1,1E-01
mmu04151:PI3K-Akt signaling pathway	36	2,65	1,1E-02	ITGB1, CSF1R, YWHAB, LAMC1, THBS1, PIK3R5, PPP2CA, COMP, CCND2, PPP2R3D, GNG5, CREB3L2, AKT1, GNG8, ITGAV, JAK3, MAGI1, MAP2K1, HSP90AA1, MAP2K2, SYK, NOS3, BAD, PRKCA, MTOR, NR4A1, COL4A4, PPP2R2D, ITGA11, COL4A5, GNB3, PKN1, FGFR3, EPHA2, TLR2, FGFR1	587	359	8941	1,527	9,7E-01	1,6E-01	1,4E-01
mmu05145:Toxoplasmosis	15	1,10	1,2E-02	ITGB1, MAP2K3, H2-EB1, BAD, H2-DMA, IL10RA, LAMC1, HSPA2, H2-AA, PIK3R5, AKT1, CCR5, HSPA1B, HSPA1A, TLR2	587	110	8941	2,077	9,8E-01	1,6E-01	1,5E-01
mmu05200:Pathways in cancer	50	3,68	1,3E-02	ITGB1, SPI1, LAMC1, CCND2, EDNRB, PIM1, AKT1, ITGAV, ELOB, JAK3, PRKCG, MAP2K1, HSP90AA1, MAP2K2, WNT5B, GSTO1, DAPK2, DAPK3, PRKCA, COL4A4, RARA, COL4A5, CSF1R, RALB, NOTCH4, PTGER3, CXCR4, ADCY2, GNG5, HMOX1, GNG8, HES1, NQO1, CDKN2B, JUN, FZD2, TXNRD3, GADD45B, JUP, GADD45A, BAD, TXNRD1, MTOR, AGT, GADD45G, NFKB2, GNAS, GNB3, FGFR3, FGFR1	587	543	8941	1,403	9,9E-01	1,6E-01	1,5E-01
mmu00230:Purine metabolism	17	1,25	1,3E-02	RRM2, ENTPD2, PDE1C, PRUNE1, NME2, GMPR, ADCY2, APRT, NME1, NT5E, PNP, GUK1, ADSSL1, PGM2, NUDT16, ADSS, PDE9A	587	133	8941	1,947	9,9E-01	1,6E-01	1,5E-01
mmu04915:Estrogen signaling pathway	17	1,25	1,4E-02	SHC2, MAP2K1, JUN, HSP90AA1, MAP2K2, SHC1, NOS3, ADCY2, HSPA2, CREB3L2, RARA, GNAS, AKT1, CTSD, HSPA1B, HSPA1A, FKBP5	587	134	8941	1,932	9,9E-01	1,7E-01	1,5E-01
mmu05140:Leishmaniasis	11	0,81	1,5E-02	ITGB1, FCGR3, H2-EB1, JUN, ITGAM, NCF1, NCF2, H2-DMA, PTPN6, H2-AA, TLR2	587	70	8941	2,394	9,9E-01	1,8E-01	1,6E-01
mmu04136:Autophagy - other	7	0,52	1,6E-02	PPP2CA, BECN1, GABARAPL2, GABARAPL1, ATG101, WIPI1, MTOR	587	32	8941	3,332	9,9E-01	1,8E-01	1,6E-01
mmu05169:Epstein-Barr virus infection	25	1,84	1,6E-02	H2-T23, H2-T22, PSMD11, PSMD14, PSMD13, H2-DMA, H2-Q4, CCND2, AKT1, HES1, JAK3, MAP2K3, JUN, H2-EB1, GADD45B, SYK, GADD45A, ADRM1, H2-AA, GADD45G, NFKB2, SEM1, NCOR2, H2-D1, TLR2	587	231	8941	1,648	1,0E+00	1,8E-01	1,6E-01
mmu05134:Legionellosis	10	0,74	1,7E-02	EEF1G, ARF2, ITGAM, RAB1B, HSPA2, SEC22B, HSPA1B, NFKB2, TLR2, HSPA1A	587	61	8941	2,497	1,0E+00	1,8E-01	1,6E-01
mmu04810:Regulation of actin cytoskeleton	24	1,77	1,7E-02	ITGB1, MAP2K1, ITGAM, MAP2K2, LIMK2, LIMK1, CXCR4, MYL12A, VAV1, SCIN, CFL2, ITGA11, PIP5K1A, PAK6, ITGAV, NCKAP1L, PIP5K1C, ARHGEF7, PAK3, FGFR3, MYH10, BCAR1, FGFR1, PAK4	587	220	8941	1,662	1,0E+00	1,8E-01	1,6E-01
mmu04213:Longevity regulating pathway - multiple species	10	0,74	1,9E-02	PRKAB2, AKT1S1, PRKAG2, AKT1, IRS2, ADCY2, HSPA2, HSPA1B, MTOR, HSPA1A	587	62	8941	2,457	1,0E+00	1,8E-01	1,7E-01
mmu04514:Cell adhesion molecules	20	1,47	1,9E-02	CD86, ITGB1, H2-T23, NTNG2, H2-EB1, H2-T22, ITGAM, H2-DMA, H2-Q4, H2-AA, PTPRF, CLDN5, OCLN, CDH3, CLDN15, CLDN23, NCAM1, ITGAV, MPZL1, H2-D1	587	174	8941	1,751	1,0E+00	1,8E-01	1,7E-01
mmu00500:Starch and sucrose metabolism	7	0,52	2,2E-02	GPI1, UGP2, GYG, PGM2, PYGL, GCK, HK1	587	34	8941	3,136	1,0E+00	2,0E-01	1,8E-01
mmu05214:Glioma	11	0,81	2,2E-02	PRKCG, SHC2, MAP2K1, MAP2K2, GADD45B, SHC1, GADD45A, AKT1, PRKCA, MTOR, GADD45G	587	74	8941	2,264	1,0E+00	2,0E-01	1,8E-01
mmu05166:Human T-cell leukemia virus 1 infection	26	1,91	2,2E-02	H2-T23, H2-T22, SPI1, ANAPC15, SRF, H2-DMA, H2-Q4, ADCY2, CCND2, CREB3L2, AKT1, MSX1, JAK3, MAP2K1, JUN, CDKN2B, H2-EB1, RANBP3, MAP2K2, ANAPC7, IL1R1, H2-AA, NFKB2, CDC16, SLC25A5, H2-D1	587	250	8941	1,584	1,0E+00	2,0E-01	1,8E-01
mmu04978:Mineral absorption	9	0,66	2,3E-02	SLC9A3, HEPHL1, ATP2B4, HMOX1, TRF, MT2, TRPM6, ATOX1, HMOX2	587	54	8941	2,539	1,0E+00	2,0E-01	1,8E-01
mmu04068:FoxO signaling pathway	16	1,18	2,4E-02	GABARAPL2, PRKAB2, CDKN2B, MAP2K1, GABARAPL1, MAP2K2, GADD45B, GADD45A, SETD7, PRKAG2, IRS2, KLF2, GADD45G, CCND2, AKT1, HOMER3	587	131	8941	1,860	1,0E+00	2,0E-01	1,8E-01
mmu04360:Axon guidance	20	1,47	2,8E-02	ITGB1, NTNG2, SEMA7A, WNT5B, UNC5B, LIMK2, LIMK1, SEMA3B, CXCR4, PRKCA, NTN1, MYL12A, PARD6B, CFL2, PAK6, PLXNB2, PAK3, EPHB4, EPHA2, PAK4	587	181	8941	1,683	1,0E+00	2,3E-01	2,0E-01
mmu04010:MAPK signaling pathway	29	2,13	2,9E-02	CSF1R, PTPRR, SRF, ARRB2, RPS6KA4, MKNK2, AKT1, MAP3K6, PRKCG, MAP2K3, DUSP4, MAP2K1, JUN, DUSP3, MAP2K2, GADD45B, GADD45A, IL1R1, PRKCA, HSPA2, DUSP6, GADD45G, NFKB2, NR4A1, FGFR3, HSPA1B, EPHA2, HSPA1A, FGFR1	587	294	8941	1,502	1,0E+00	2,3E-01	2,1E-01
mmu03050:Proteasome	8	0,59	3,2E-02	SEM1, PSMD11, PSMB5, PSMD14, PSMD13, PSMB1, ADRM1, PSMA7	587	47	8941	2,593	1,0E+00	2,4E-01	2,2E-01

Table S11. KEGG pathway analysis of all genes that are downregulated in hearts of AAV9-Luc but not AAV9-Tfeb TAC treated mice

Term	Count	%	PValue	Genes	List Tota	Pop Hits	Pop Tota	Fold Enrichmer	Bonferror	Benjamir	FDR
mmu01100:Metabolic pathways	40	22,86	8,9E-09	ACADVL, GSTP2, GALNT15, ATP5A1, MAT2B, OPLAH, COX7C, ACACB, ENO3, AGPAT3, PTS, LDHB, ADH1, CYP26B1, AUH, NNT, CYP2B10, ME3, HMGCS2, MLYCD, ACSS1, NDUFV1, CAR4, BCKDHA, MDH1, GOT1, EPHX2, ACSL6, SDHC, COX6C, FAH, GNMT, GSTA3, NDUFAB1, ACOT2, KYAT3, NDUFS2, LPIN1, BCAT2, CDS2	89	1609	8941	2,497	2,0E-06	2,0E-06	1,8E-06
mmu05208:Chemical carcinogenesis - reactive oxygen species	11	6,29	6,0E-05	GSTA3, AKT2, EPHX2, ATP5A1, NDUFAB1, NDUFS2, SDHC, COX6C, SOD2, COX7C, NDUFV1	89	222	8941	4,978	1,3E-02	4,8E-03	4,5E-03
mmu00620:Pyruvate metabolism	6	3,43	6,6E-05	LDHB, ADH1, MDH1, ME3, ACSS1, ACACB	89	44	8941	13,699	1,4E-02	4,8E-03	4,5E-03
mmu00270:Cysteine and methionine metabolism	6	3,43	1,6E-04	LDHB, MDH1, GOT1, KYAT3, MAT2B, BCAT2	89	53	8941	11,373	3,5E-02	8,4E-03	7,9E-03
mmu00640:Propanoate metabolism	5	2,86	2,3E-04	BCKDHA, LDHB, MLYCD, ACSS1, ACACB	89	31	8941	16,203	4,8E-02	8,4E-03	7,9E-03
mmu03320:PPAR signaling pathway	7	4,00	2,3E-04	ACSL6, PLIN4, LPL, APOA1, ME3, HMGCS2, CD36	89	89	8941	7,901	4,9E-02	8,4E-03	7,9E-03
mmu04714:Thermogenesis	10	5,71	4,3E-04	ATP5A1, NDUFAB1, ACSL6, PRKAG1, NDUFS2, SDHC, SLC25A20, COX6C, COX7C, NDUFV1	89	231	8941	4,349	8,9E-02	1,3E-02	1,2E-02
mmu04932:Non-alcoholic fatty liver disease	8	4,57	8,4E-04	AKT2, NDUFAB1, PRKAG1, NDUFS2, SDHC, COX6C, COX7C, NDUFV1	89	156	8941	5,152	1,7E-01	2,3E-02	2,1E-02
mmu05415:Diabetic cardiomyopathy	9	5,14	1,1E-03	AKT2, ATP5A1, NDUFAB1, NDUFS2, SDHC, CD36, COX6C, COX7C, NDUFV1	89	211	8941	4,285	2,1E-01	2,6E-02	2,4E-02
mmu04146:Peroxisome	6	3,43	1,5E-03	EPHX2, PEX6, PXMP2, ACSL6, MLYCD, SOD2	89	86	8941	7,009	2,9E-01	3,4E-02	3,1E-02
mmu00190:Oxidative phosphorylation	7	4,00	2,1E-03	ATP5A1, NDUFAB1, NDUFS2, SDHC, COX6C, COX7C, NDUFV1	89	135	8941	5,209	3,7E-01	4,2E-02	3,9E-02
mmu04936:Alcoholic liver disease	7	4,00	2,6E-03	ADH1, ACADVL, AKT2, PRKAG1, MLYCD, LPIN1, ACACB	89	141	8941	4,987	4,3E-01	4,7E-02	4,4E-02
mmu04710:Circadian rhythm	4	2,29	3,1E-03	PER2, PER1, CRY2, PRKAG1	89	30	8941	13,395	4,9E-01	5,2E-02	4,9E-02
mmu05012:Parkinson disease	9	5,14	4,3E-03	HSPA5, ATP5A1, NDUFAB1, NDUFS2, SDHC, COX6C, COX7C, NDUFV1, SLC18A2	89	264	8941	3,425	6,1E-01	6,8E-02	6,4E-02
mmu04920:Adipocytokine signaling pathway	5	2,86	5,2E-03	AKT2, ACSL6, PRKAG1, CD36, ACACB	89	71	8941	7,075	6,8E-01	7,1E-02	6,6E-02
mmu00982:Drug metabolism - cytochrome P450	5	2,86	5,2E-03	ADH1, GSTP2, GSTA3, FMO1, FMO2	89	71	8941	7,075	6,8E-01	7,1E-02	6,6E-02
mmu01200:Carbon metabolism	6	3,43	6,7E-03	MDH1, GOT1, ME3, SDHC, ACSS1, ENO3	89	121	8941	4,982	7,7E-01	8,6E-02	8,1E-02
mmu00071:Fatty acid degradation	4	2,29	1,4E-02	ADH1, ACADVL, ECI1, ACSL6	89	52	8941	7,728	9,6E-01	1,8E-01	1,6E-01
mmu05020:Prion disease	8	4,57	1,6E-02	HSPA5, ATP5A1, NDUFAB1, NDUFS2, SDHC, COX6C, COX7C, NDUFV1	89	268	8941	2,999	9,7E-01	1,9E-01	1,7E-01
mmu00280:Valine, leucine and isoleucine degradation	4	2,29	1,8E-02	BCKDHA, AUH, HMGCS2, BCAT2	89	57	8941	7,050	9,8E-01	2,0E-01	1,9E-01
mmu04964:Proximal tubule bicarbonate reclamation	3	1,71	1,9E-02	MDH1, ATP1B2, CAR4	89	22	8941	13,699	9,9E-01	2,0E-01	1,9E-01
mmu00010:Glycolysis / Gluconeogenesis	4	2,29	2,8E-02	LDHB, ADH1, ACSS1, ENO3	89	67	8941	5,998	1,0E+00	2,6E-01	2,5E-01
mmu05014:Amyotrophic lateral sclerosis	9	5,14	2,8E-02	HSPA5, ATP5A1, NDUFAB1, ANXA11, NDUFS2, SDHC, COX6C, COX7C, NDUFV1	89	369	8941	2,450	1,0E+00	2,6E-01	2,5E-01
mmu05016:Huntington disease	8	4,57	2,9E-02	ATP5A1, NDUFAB1, NDUFS2, SDHC, COX6C, SOD2, COX7C, NDUFV1	89	302	8941	2,661	1,0E+00	2,6E-01	2,5E-01
mmu05010:Alzheimer disease	9	5,14	3,4E-02	AKT2, ATP5A1, NDUFAB1, NDUFS2, LPL, SDHC, COX6C, COX7C, NDUFV1	89	383	8941	2,361	1,0E+00	3,0E-01	2,8E-01
mmu04152:AMPK signaling pathway	5	2,86	3,5E-02	AKT2, PRKAG1, MLYCD, CD36, ACACB	89	126	8941	3,987	1,0E+00	3,0E-01	2,8E-01
mmu01230:Biosynthesis of amino acids	4	2,29	4,3E-02	GOT1, MAT2B, ENO3, BCAT2	89	79	8941	5,087	1,0E+00	3,5E-01	3,2E-01

Table S12. KEGG pathway analysis of all genes that are downregulated in hearts of AAV9-Luc and AAV9-Tfeb TAC treated mice

Term	Count	%	PValue	Genes	List Tota	Pop Hits	Pop Tota	Fold Enrichmen	Bonferron	Benjamin	FDR
mmu01100:Metabolic pathways	102	14,23	2,5E-11	ALDH1L1, ACAA2, PANK1, ACSM5, OGDHL, HNMT, ALDH1L2, PPAT, NAMPT, CAR14, CHAC2, HADH, NUDT12, MCCC2, GSTK1, DGAT2, GPT2, ENTPD5, MCCC1, DHDH, COX6B2, SDHA, GANC, GCAT, CYP2U1, ACOT1, SUCLG2, SUCLG1, AMY1, PFKFB1, GCDH, MAOB, GSTT2, ATP5J, GSTT1, ALOX12, PLA2G5, MTMR4, COX7A1, MAN2A2, LDHD, PRODH, FDFT1, GGT5, GSTM1, SYNJ2, ACADSB, ALDH6A1, GGCX, ADI1, GSTM7, ACY1, MOGAT2, AMD1, MTR, GPT, PFAS, DHTKD1, NADK2, ALDH2, AOX3, DBT, PDE4A, AOX1, ACADM, IDH3A, PDE4D, PLA2G4B, BCKDHB, PDE4C, AMT, SORD, PGM2L1, INMT, HADHB, HADHA, ALDH5A1, PCCA, IVD, PDE5A, NDUFS1, ALDOB, FBP2, ALDH9A1, ST6GALNAC6, DDC, ACSS3, ADCY6, SELENBP1, SELENBP2, ALOX5, FKTN, DUT, NDUFA5, AGL, GATB, DHODH, ALDH4A1, NT5C1A, CTH, GALM, PFKM	301	1609	8941	1,883	6,7E-09	3,8E-09	3,8E-09
mmu00280:Valine, leucine and isoleucine degradation	17	2,37	2,9E-11	MCCC2, ACAA2, MCCC1, BCKDHB, ACADSB, HADHB, HADHA, ALDH6A1, PCCA, ALDH2, IVD, AOX3, DBT, AOX1, ACADM, HADH, ALDH9A1	301	57	8941	8,859	7,7E-09	3,8E-09	3,8E-09
mmu00071:Fatty acid degradation	11	1,53	7,1E-06	HADHB, GCDH, HADHA, ACAA2, CPT2, ALDH2, CYP2U1, ACADM, HADH, ACADSB, ALDH9A1	301	52	8941	6,284	1,9E-03	4,7E-04	4,6E-04
mmu00380:Tryptophan metabolism	11	1,53	7,1E-06	GCDH, HADHA, DDC, MAOB, ALDH2, AOX3, AOX1, HADH, INMT, ALDH9A1, DHTKD1	301	52	8941	6,284	1,9E-03	4,7E-04	4,6E-04
mmu00640:Propanoate metabolism	8	1,12	5,9E-05	HADHA, ACSS3, ALDH6A1, PCCA, DBT, BCKDHB, SUCLG2, SUCLG1	301	31	8941	7,666	1,5E-02	3,1E-03	3,1E-03
mmu04020:Calcium signaling pathway	20	2,79	4,4E-04	CHRM2, PTGFR, CAMK2A, ATP2A2, ADRB1, AVPR1A, ADRA1B, ADRA1A, CACNA1H, MYLK3, MYLK, MYLK4, FGF16, P2RX6, PLN, PHKG1, P2RX1, HRC, CASQ1, FGF10	301	240	8941	2,475	1,1E-01	2,0E-02	1,9E-02
mmu01200:Carbon metabolism	13	1,81	7,3E-04	GPT2, AMT, OGDHL, GPT, SDHA, ALDH6A1, PCCA, SUCLG2, SUCLG1, ALDOB, PFKM, FBP2, IDH3A	301	121	8941	3,191	1,8E-01	2,8E-02	2,7E-02
mmu04261:Adrenergic signaling in cardiomyocytes	13	1,81	5,0E-03	CAMK2A, ATP2A2, CACNA2D2, ADRB1, ADRA1B, ADRA1A, ADCY6, CACNB2, PLN, RPS6KA5, PPP2R2C, TNNI3, MYH6	301	152	8941	2,541	7,4E-01	1,7E-01	1,6E-01
mmu04024:cAMP signaling pathway	16	2,23	7,1E-03	CHRM2, PTCH1, PDE4D, CAMK2A, PDE4C, ATP2A2, ADRB1, ADCY6, CRHR2, PLN, ADORA1, PDE4A, TNNI3, CNGA3, PPARA, DRD2	301	220	8941	2,160	8,5E-01	2,1E-01	2,1E-01
mmu00982:Drug metabolism - cytochrome P450	8	1,12	9,5E-03	GSTK1, GSTM1, MAOB, AOX3, AOX1, GSTT2, GSTT1, GSTM7	301	71	8941	3,347	9,2E-01	2,5E-01	2,5E-01
mmu00760:Nicotinate and nicotinamide metabolism	6	0,84	1,1E-02	NADK2, NT5C1A, NAMPT, AOX3, AOX1, NUDT12	301	41	8941	4,347	9,5E-01	2,8E-01	2,7E-01
mmu04022:cGMP-PKG signaling pathway	13	1,81	1,4E-02	ATP2A2, ADRB1, ADRA1B, ADRA2B, ADRA1A, ADCY6, MYLK3, MYLK, MYLK4, PLN, ADORA1, PDE5A, MYH6	301	173	8941	2,232	9,7E-01	2,8E-01	2,8E-01
mmu04921:Oxytocin signaling pathway	12	1,67	1,4E-02	CACNB2, KCNJ5, KCNJ12, PLA2G4B, CAMK2A, CACNA2D2, KCNJ2, ADCY6, MYLK3, MYLK, KCNJ3, MYLK4	301	153	8941	2,330	9,7E-01	2,8E-01	2,8E-01
mmu00062:Fatty acid elongation	5	0,70	1,5E-02	HADHB, HADHA, ACAA2, ACOT1, HADH	301	29	8941	5,121	9,8E-01	2,9E-01	2,8E-01
mmu01230:Biosynthesis of amino acids	8	1,12	1,6E-02	ACY1, GPT2, CTH, MTR, GPT, ALDOB, PFKM, IDH3A	301	79	8941	3,008	9,9E-01	2,9E-01	2,8E-01
mmu01212:Fatty acid metabolism	7	0,98	1,7E-02	HADHB, HADHA, ACAA2, CPT2, ACADM, HADH, ACADSB	301	62	8941	3,354	9,9E-01	2,9E-01	2,8E-01
mmu00020:Citrate cycle (TCA cycle)	5	0,70	2,1E-02	SUCLG2, SUCLG1, OGDHL, SDHA, IDH3A	301	32	8941	4,641	1,0E+00	3,3E-01	3,3E-01
mmu00670:One carbon pool by folate	4	0,56	2,4E-02	ALDH1L1, AMT, MTR, ALDH1L2	301	19	8941	6,254	1,0E+00	3,5E-01	3,4E-01
mmu05415:Diabetic cardiomyopathy	14	1,95	2,5E-02	NDUFA5, CAMK2A, ATP2A2, ATP5J, SLC2A4, SDHA, COX6B2, COX7A1, PLN, CPT2, TBC1D4, TNNI3, NDUFS1, PPARA	301	211	8941	1,971	1,0E+00	3,5E-01	3,4E-01
mmu04260:Cardiac muscle contraction	8	1,12	2,7E-02	CACNB2, HRC, ATP2A2, CACNA2D2, TNNI3, COX6B2, COX7A1, MYH6	301	87	8941	2,731	1,0E+00	3,5E-01	3,4E-01
mmu01210:2-Oxocarboxylic acid metabolism	4	0,56	2,8E-02	ACY1, GPT2, GPT, IDH3A	301	20	8941	5,941	1,0E+00	3,5E-01	3,5E-01
mmu00051:Fructose and mannose metabolism	5	0,70	3,1E-02	PFKFB1, SORD, ALDOB, PFKM, FBP2	301	36	8941	4,126	1,0E+00	3,8E-01	3,7E-01
mmu00480:Glutathione metabolism	7	0,98	3,3E-02	GGT5, GSTK1, GSTM1, GSTT2, GSTT1, CHAC2, GSTM7	301	72	8941	2,888	1,0E+00	3,8E-01	3,7E-01
mmu00330:Arginine and proline metabolism	6	0,84	3,4E-02	ALDH4A1, MAOB, ALDH2, AMD1, PRODH, ALDH9A1	301	54	8941	3,300	1,0E+00	3,8E-01	3,7E-01
mmu05208:Chemical carcinogenesis - reactive oxygen species	14	1,95	3,6E-02	GSTM1, NDUFA5, ATP5J, GSTT2, GSTT1, SDHA, FOXO3, COX6B2, COX7A1, AS3MT, NDUFS1, GSTM7, SLC26A6, MAP3K5	301	222	8941	1,873	1,0E+00	3,8E-01	3,7E-01
mmu05414:Dilated cardiomyopathy	8	1,12	3,8E-02	CACNB2, PLN, ATP2A2, CACNA2D2, ADRB1, TNNI3, MYH6, ADCY6	301	94	8941	2,528	1,0E+00	3,8E-01	3,7E-01
mmu04010:MAPK signaling pathway	17	2,37	3,9E-02	MAP4K2, ANGPT1, MAX, PLA2G4B, IGF2, CACNA2D2, RASGRP2, CACNA1H, DUSP7, FGF16, CACNB2, RPS6KA5, NTF3, RAPGEF2, MAP3K13, MAP3K5, FGF10	301	294	8941	1,718	1,0E+00	3,8E-01	3,7E-01
mmu00250:Alanine, aspartate and glutamate metabolism	5	0,70	4,1E-02	ALDH4A1, ALDH5A1, GPT2, PPAT, GPT	301	39	8941	3,808	1,0E+00	3,8E-01	3,8E-01
mmu04713:Circadian entrainment	8	1,12	4,6E-02	KCNJ5, PER3, RPS6KA5, CAMK2A, NOS1AP, CACNA1H, ADCY6, KCNJ3	301	98	8941	2,425	1,0E+00	4,2E-01	4,1E-01
mmu04919:Thyroid hormone signaling pathway	9	1,26	4,8E-02	PLN, RXRA, THRB, TBC1D4, ATP2A2, SLC16A10, PFKM, MYH6, MED12L	301	120	8941	2,228	1,0E+00	4,3E-01	4,2E-01

Table S13. KEGG pathway analysis of all genes that are downregulated in hearts of AAV9-Tfeb but not AAV9-Luc TAC treated mice

Term	Count	%	PValue	Genes	List Tota	Pop Hits	Pop Tota	Fold Enrichmen	Bonferron	Benjamin	FDR
mmu05168:Herpes simplex virus 1 infection	48	4,21	1,9E-06	H2-T24, ZFP788, ZFP128, ZFP763, ZFP961, ZFP109, ZFP867, ZFP866, ZFP947, ZFP60, CFP, ZFP729B, ZFP809, ZFP78, ZFP12, ZFP14, A430033K04RIK, ZFP874B, ZFP74, ZFP874A, ZFP180, ZFP120, ZFP141, ZFP266, ZFP420, ZFP386, ZFP661, ZFP799, ZFP235, ZFP950, ZFP715, ZFP317, ZFP811, TSC1, ZFP958, ZFP780B, ZFP68, OAS2, ZFP65, ZFP273, ZFP597, ZFP113, SRSF7, ZFP112, ZFP398, ZFP870, ZFP551, ZFP275	449	459	8941	2,082	5,7E-04	5,7E-04	5,7E-04
mmu04022:cGMP-PKG signaling pathway	20	1,75	1,0E-03	OPRD1, ROCK1, IRS1, PDE2A, ATP2A3, NFATC3, ATP2A1, ATP1A2, ADCY1, ADCY8, PPP3CB, RGS2, CREB1, ADCY9, PDE3A, VDAC3, CACNA1S, RAF1, MYL9, GTF2I	449	173	8941	2,302	2,7E-01	1,6E-01	1,6E-01
mmu04935:Growth hormone synthesis, secretion and action	15	1,32	1,9E-03	STAT5A, STAT5B, IRS1, ADCY1, MAPK14, ADCY8, SSTR3, MAPK9, CREB1, ADCY9, EP300, CACNA1S, RAF1, SOS2, MAP2K6	449	116	8941	2,575	4,4E-01	1,9E-01	1,9E-01
mmu04912:GnRH signaling pathway	11	0,96	1,4E-02	CAMK2B, MAPK9, ADCY9, CACNA1S, ADCY1, MAPK14, ADCY8, RAF1, ELK1, SOS2, MAP2K6	449	90	8941	2,434	9,9E-01	5,5E-01	5,5E-01
mmu05205:Proteoglycans in cancer	19	1,67	1,4E-02	CAMK2B, DDX5, ROCK1, CAV1, WNT5A, ANK2, MAPK14, ELK1, FGF2, VTN, ERBB3, PDCD4, GPC3, KDR, DROSHA, HPSE, PPP1R12B, RAF1, SOS2	449	205	8941	1,846	9,9E-01	5,5E-01	5,5E-01
mmu04919:Thyroid hormone signaling pathway	13	1,14	1,7E-02	NOTCH2, NCOA1, MED1, NCOA2, HDAC2, ATP2A3, ATP2A1, ATP1A2, SIN3A, EP300, RAF1, PLCD4, RXRG	449	120	8941	2,157	9,9E-01	5,5E-01	5,5E-01
mmu05414:Dilated cardiomyopathy	11	0,96	1,9E-02	RYR2, ADCY9, SGCA, MYL2, ITGA2B, ATP2A3, ATP2A1, CACNA1S, ITGB6, ADCY1, ADCY8	449	94	8941	2,330	1,0E+00	5,5E-01	5,5E-01
mmu04261:Adrenergic signaling in cardiomyocytes	15	1,32	2,0E-02	CAMK2B, RYR2, ATP2A3, ATP2A1, ATP1A2, PPP2R5D, ADCY1, PPP2R3A, MAPK14, ADCY8, CREB1, ADCY9, MYL2, CACNA1S, SCN4B	449	152	8941	1,965	1,0E+00	5,5E-01	5,5E-01
mmu00020:Citrate cycle (TCA cycle)	6	0,53	2,0E-02	CS, SUCLA2, IDH1, IDH3B, DLD, FH1	449	32	8941	3,734	1,0E+00	5,5E-01	5,5E-01
mmu04921:Oxytocin signaling pathway	15	1,32	2,1E-02	CAMK2B, RYR2, ROCK1, NFATC3, ADCY1, ADCY8, ELK1, PPP3CB, RGS2, ADCY9, CAMK4, CACNA1S, PPP1R12B, RAF1, MYL9	449	153	8941	1,952	1,0E+00	5,5E-01	5,5E-01
mmu04010:MAPK signaling pathway	24	2,11	2,2E-02	EGF, RASGRF2, NFATC3, PDGFA, MAPK14, ELK1, FGF2, DUSP16, RASGRP3, MAPK9, PPP3CB, DUSP10, MAPKAPK3, FGF9, ERBB3, KITL, RPS6KA2, MAP3K20, KDR, CACNA1S, TEK, RAF1, SOS2, MAP2K6	449	294	8941	1,626	1,0E+00	5,5E-01	5,5E-01
mmu04024:cAMP signaling pathway	19	1,67	2,7E-02	CAMK2B, HCN4, RYR2, ROCK1, ATP2A3, ATP2A1, ATP1A2, ADCY1, ADCY8, MAPK9, CREB1, ADCY9, CAMK4, PPP1R1B, PDE3A, EP300, CACNA1S, RAF1, MYL9	449	220	8941	1,720	1,0E+00	5,5E-01	5,5E-01
mmu04540:Gap junction	10	0,88	2,8E-02	ADCY9, EGF, TUBB1, PDGFA, ADCY1, ADCY8, RAF1, SOS2, TUBA4A, TUBA8	449	86	8941	2,315	1,0E+00	5,5E-01	5,5E-01
mmu04911:Insulin secretion	10	0,88	2,8E-02	CAMK2B, CHRM3, RYR2, CREB1, ADCY9, ATP1A2, KCNN2, CACNA1S, ADCY1, ADCY8	449	86	8941	2,315	1,0E+00	5,5E-01	5,5E-01
mmu04020:Calcium signaling pathway	20	1,75	3,2E-02	CAMK2B, CHRM3, RYR2, EGF, ATP2A3, PDGFA, ATP2A1, ADCY1, ADCY8, FGF2, ITPKB, PPP3CB, ADCY9, FGF9, ERBB3, CAMK4, KDR, VDAC3, CACNA1S, PLCD4	449	240	8941	1,659	1,0E+00	5,5E-01	5,5E-01
mmu05200:Pathways in cancer	38	3,33	3,3E-02	NOTCH2, CAMK2B, HDAC2, ROCK1, GSTP1, ITGA2B, PDGFA, ADCY1, ADCY8, ELK1, FGF2, HSP90B1, RASGRP3, MAPK9, BCL2L11, FGF9, HEY1, HEY2, EP300, RXRG, STAT5A, NCOA1, STAT5B, EGLN3, LAMB3, EGF, WNT5A, AXIN2, MLH1, FH1, DDB2, AR, CXCL12, ADCY9, KITL, IL2RA, RAF1, SOS2	449	543	8941	1,394	1,0E+00	5,5E-01	5,5E-01
mmu04660:T cell receptor signaling pathway	11	0,96	3,3E-02	MAPK9, PPP3CB, DLG1, TEC, GRAP2, NFATC3, CTLA4, PRKCQ, MAPK14, RAF1, SOS2	449	103	8941	2,127	1,0E+00	5,5E-01	5,5E-01
mmu02010:ABC transporters	7	0,61	3,8E-02	ABCA1, ABCA2, ABCD2, ABCC5, ABCA7, ABCB1A, ABCB1B	449	50	8941	2,788	1,0E+00	5,5E-01	5,5E-01
mmu04360:Axon guidance	16	1,40	3,8E-02	CAMK2B, SEMA5B, NRP1, EPHA4, EPHA7, ROCK1, TRPC3, WNT5A, NFATC3, PRKCZ, PPP3CB, CXCL12, PARD3, RAF1, MYL9, EPHB3	449	181	8941	1,760	1,0E+00	5,5E-01	5,5E-01
mmu00563:Glycosylphosphatidylinositol (GPI)-anchor biosynthesis	5	0,44	3,9E-02	PIGU, PGAP1, PIGO, PIGG, PIGV	449	26	8941	3,829	1,0E+00	5,5E-01	5,5E-01
mmu00310:Lysine degradation	8	0,70	4,0E-02	COLGALT2, KMT2A, SETDB1, KMT2C, PRDM2, DLD, EZH1, AASS	449	64	8941	2,489	1,0E+00	5,5E-01	5,5E-01
mmu04015:Rap1 signaling pathway	18	1,58	4,0E-02	EGF, ITGA2B, PDGFA, ADCY1, MAPK14, ADCY8, PRKCZ, FGF2, RAP1GAP, RASGRP3, ADCY9, FGF9, KITL, PARD3, KDR, TEK, RAF1, MAP2K6	449	214	8941	1,675	1,0E+00	5,5E-01	5,5E-01
mmu05166:Human T-cell leukemia virus 1 infection	20	1,75	4,5E-02	H2-T24, STAT5A, NRP1, STAT5B, NFATC3, ADCY1, ADCY8, ELK1, MAPK9, DLG1, PPP3CB, CREB1, XPO1, ADCY9, PTTG1, CHEK2, IL2RA, VDAC3, EP300, ATM	449	250	8941	1,593	1,0E+00	5,6E-01	5,6E-01
mmu04510:Focal adhesion	17	1,49	4,5E-02	ROCK1, LAMB3, EGF, CAV1, ITGA2B, PDGFA, ARHGAP5, ELK1, VTN, MAPK9, MYL2, KDR, ITGB6, PPP1R12B, RAF1, MYL9, SOS2	449	201	8941	1,684	1,0E+00	5,6E-01	5,6E-01
mmu04720:Long-term potentiation	8	0,70	4,9E-02	CAMK2B, PPP3CB, RPS6KA2, CAMK4, EP300, ADCY1, ADCY8, RAF1	449	67	8941	2,378	1,0E+00	5,8E-01	5,8E-01
mmu04810:Regulation of actin cytoskeleton	18	1,58	5,0E-02	CYFIP2, CHRM3, ROCK1, EGF, ITGA2B, PDGFA, IQGAP2, ACTR3B, FGF2, CXCL12, FGF9, MYL2, SPATA13, ITGB6, PPP1R12B, RAF1, MYL9, SOS2	449	220	8941	1,629	1,0E+00	5,8E-01	5,8E-01

Table S14. Primers for generation of AAV9-Tfeb cDNA expression plasmids.

**Name**	**Sequence**
Mm_Tfeb_XbaI_forward	5’-GCT CTA GAA TGG CTC AGC TCG CTC AG-3’
Mm_Tfeb_XbaI_reverse	5’-GCT CTA GAT CAC AGA ACA TCA CCC TCC TC-3’

Tfeb indicates transcription factor EB; XbaI indicates the restriction enzyme used.

Table S15. Primer pairs for real time RT-PCR.

Gene	Primer sequence
**Mm_*Atg101*_forward**	5’-CCG GTC AGA ATT CTC GGT-3’
**Mm_*Atg101*_reverse**	5’-GCA AGA CAC AGG ATA CTT CAC C-3’
**Mm_*Atp6v1h*_forward**	5-GCT CAC GAT GTT GGA GAA TAT GT-3’
**Mm_*Atp6v1h*_reverse**	5’-GCA TGT GGT TCA TCA CCA ACT-3’
**Mm_*Becn1*_forward**	5’-AGG ATG GTG TCT CTC GAA GATT -3'
**Mm_*Becn1*_reverse**	5’-GAT CAG AGT GAA GCT ATT AGC ACT TTC-3’
**Mm_Col1a1_forward**	5’-TGT AAA CAC CCC AGC GAA GAA-3’
**Mm_Col1a1_reverse**	5’-CTG AGT TGC CAT TTC CTT GGA-3’
**Mm_Col3a1_forward**	5’-CTC ACC CTT CTT CAT CCC ACT CTT A-3’
**Mm_Col3a1_reverse**	5’-ACA TGG TTC TGG CTT CCA GAC AT-3’
**Mm_*Ctgf*_forward**	5`-TTC CCG AGA AGG GTC AAG CT-3`
**Mm_*Ctgf*_reverse**	5`-TTG GGT CTG GGC CAA ATG T-3`
**Mm_*Ctsd*_forward**	5’-CAT TGC AAG ATA CTT GAC ATA GCC-3'
**Mm_*Ctsd*_reverse**	5’-CGT TCT TCA CAT AGG TGC TGG-3'
**Mm_*Ctsl*_forward**	5’-CAA ATA AGA ATA AAT ATT GGC TTG TCA-3'
**Mm_*Ctsl*_reverse**	5’-TTT GAT GTA GCC TTC CAT ACC C-3'
**Mm_*Gabarapl2*_forward**	5’-AGT CCC GCC TGT GGA GTA G-3’
**Mm_*Gabarapl2*_reverse**	5’-AGC GAC CAC AAC AAC AAC G-3’
**Mm_*Ipo8*_forward**	5’-GAG AAG ATG CCG AGT GCT ATG-3'
**Mm_*Ipo8*_reverse**	5’-GAC TTG CTC CAG TGT ATG GAA C-3'
**Mm_*Map1lc3b*_forward**	5’-GAC CAG CAC CCC AGT AAG AT-3'
**Mm_*Map1lc3b*_reverse**	5’-TGG GAC CAG AAA CTT GGT CT-3'
**Mm_*Mrpl13*_forward**	5’-ACA ATG ATG CAA AGG CTG C-3'
**Mm_*Mrpl13*_reverse**	5’-CGT GGC TGA GGA AGC TCT T-3'
**Mm_*Myh6*_forward**	5`-GCC AAG ACT GTC CGG AAT GA-3`
**Mm_*Myh6*_reverse**	5`-TGG AAG ATC ACC CGG GAC TT-3`
**Mm_*Myh7*_forward**	5`-CAA AGG CAA GGC AAA GAA AG-3`
**Mm_*Myh7*_reverse**	5`-TCA CCC CTG GAG ACT TTG TC-3`
**Mm_*Nppa*_forward**	5`-GGG GGT AGG ATT GAC AGG AT-3`
**Mm_*Nppa*_reverse**	5`-ACA CAC CAC AAG GGC TTA GG-3`
**Mm_*Nppb*_forward**	5`-GCA CAA GAT AGA CCG GAT CG-3`

**Mm_*Nppb*_reverse**	5`-CTT CAA AGG TGG TCC CAG AG-3`
**Mm_*Pgk1*_forward**	5’-CAG ACA AGA TCC AGC TGA TCA-3'
**Mm_*Pgk1*_reverse**	5’-CTT CTT CAT CAT ACA GAG ATG TGC-3'
**Mm_*Sqstm1*_forward**	5’-AGA CCC CTC ACA GGA AGG AC-3'
**Mm_*Sqstm1*_reverse**	5’-CAT CTG GGA GAG GGA CTC AA-3'
**Mm_*Tecpr1*_forward**	5’-TAT GTG TGC TCC AGT GAT GTA CC-3'
**Mm_*Tecpr1*_reverse**	5’-CTC GGC AGG AGC TTC TCA-3'
**Mm_*Tfeb*_forward**	5’-GAG CTG GGA ATG CTG ATC C-3'
**Mm_*Tfeb*_reverse**	5’-CTT GAG GAT GGT GCC TTT GT-3'
**Mm_*Wipi1*_forward**	5’-CCT ATC CTG GAA GCC AGA GTA C-3'
**Mm_*Wipi1*_reverse**	5’-CGA GGA GTT GAA GGT GAT AGC-3'

Atg101 indicates autophagy related 101; *Atp6v1h*, ATPase, H^+ transporting, lysosomal V1 subunit H; *Becn1*, Beclin-1; *Ctgf*, connective tissue growth factor; *Ctsd*, Cathepsin D; *Ctsl*, Cathepsin L; *Gabarapl2*, gamma-aminobutyric acid A receptor-associated protein-like 2; *Nppa*, natriuretic peptide A; *Nppb*, B-type natriuretic factor; *Myh*, myosin heavy chain; *Ipo8*, Importin 8; *Map1lc3b*, microtubule-associated proteins 1A/1B light chain 3B; *Mrpl13*, mitochondrial ribosomal protein L13; *Pgk1*, Phosphoglycerate Kinase 1; *Sqstm1*/p62, Sequestosome 1; *Tecpr1*, tectonin beta-propeller repeat containing 1; *Tfeb*, Transcription factor EB; *Wipi1*, WD repeat domain phosphoinositide-interacting protein 1.

Figure S1. Principal component analysis (PCA).

Figure S2. (a-c) Venn Diagrams showing the number of genes that were differentially regulated (**A**; adjusted p-value<0.05), upregulated (**b**; adjusted p-value<0.05) or downregulated (**c**; adjusted p-value<0.05) in hearts of TAC-treated AAV9-Luc or AAV9-Tfeb mice as compared to the respective sham group. (**d**) Direct comparison of gene ontology (GO) term analysis of genes that were upregulated (left panels) or downregulated (right panels) in TAC treated AAV9-Luc and AAV9-Tfeb hearts. BP indicates biological process; MF, molecular function.

Figure S3. Gene ontology (GO) term analysis of genes that were only upregulated (left panels) or downregulated (right panels) in TAC treated AAV9-Tfeb but not AAV9-Luc hearts. BP indicates biological process; MF, molecular function.

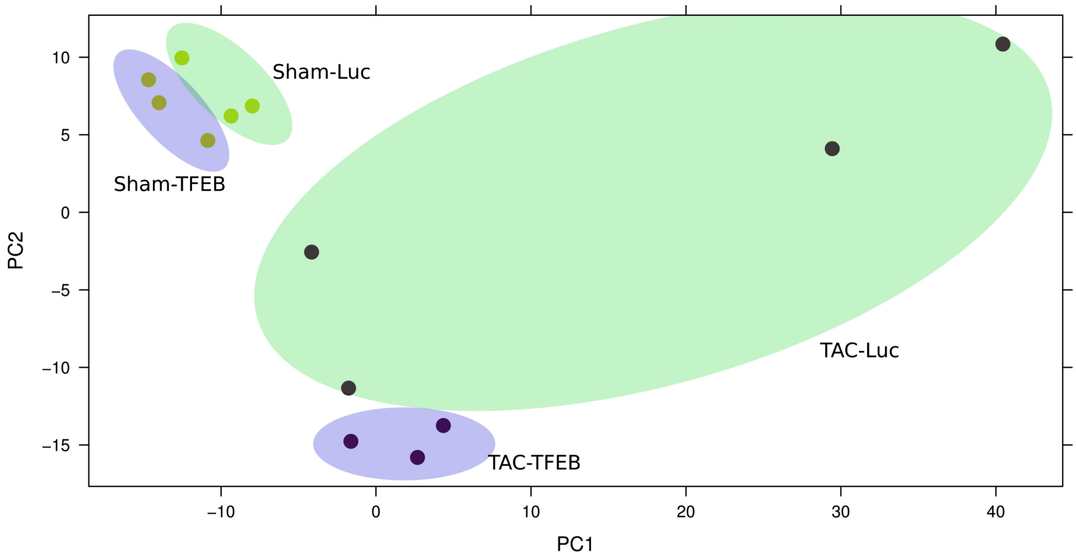

Supplementary Figure 1

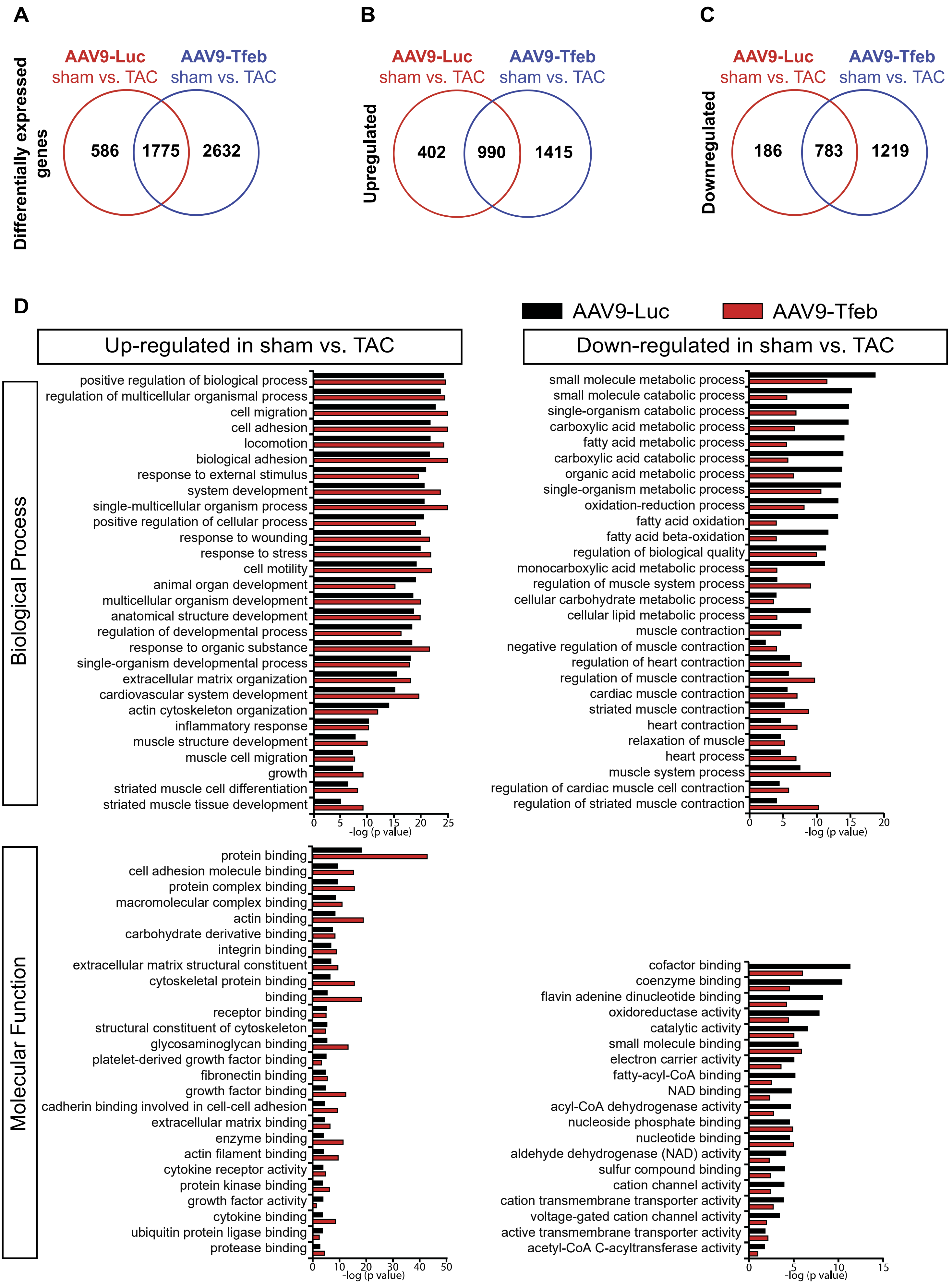

Supplementary Figure 2

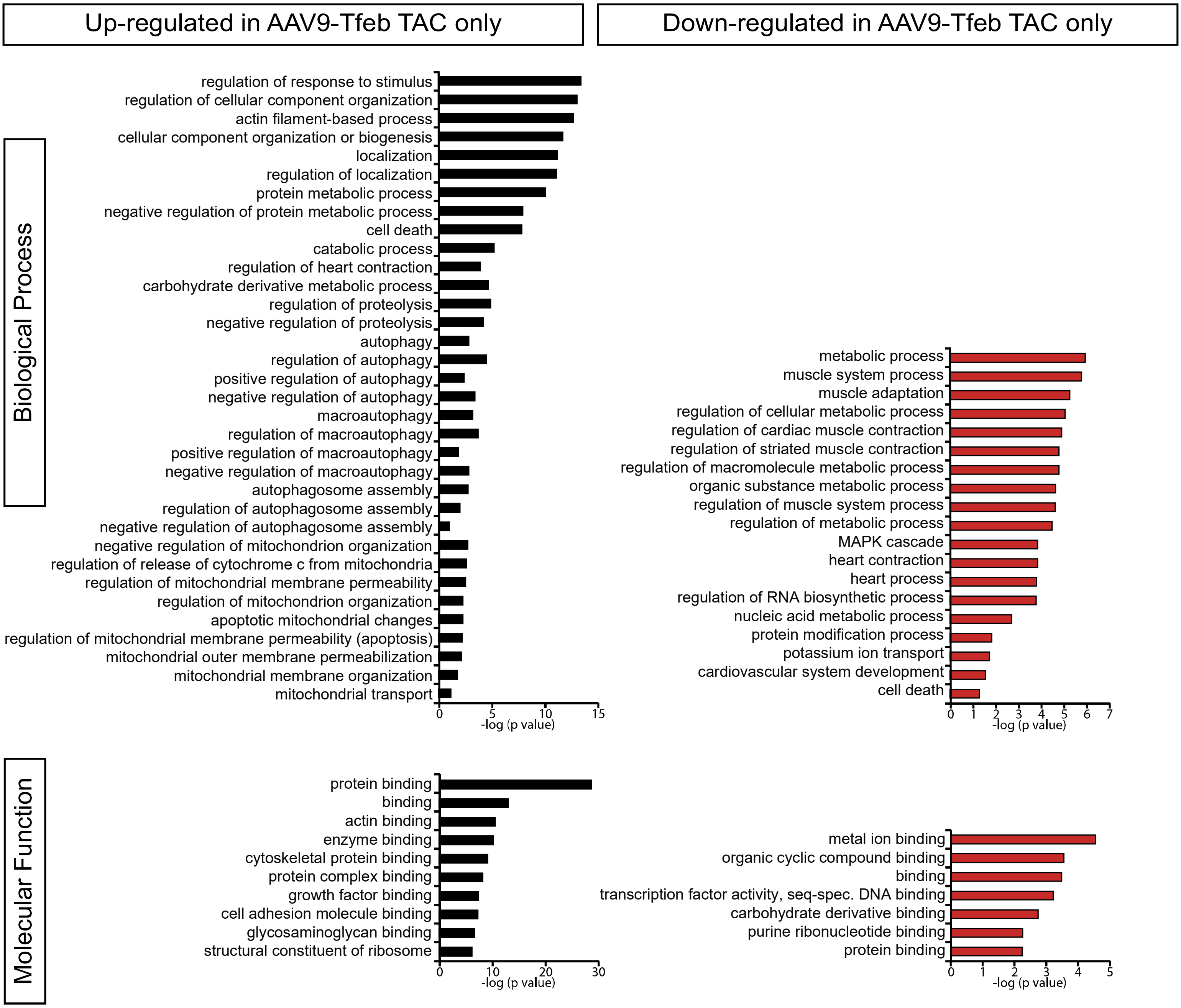

Supplementary Figure 3
